# Histone deacetylase 3 controls lung alveolar macrophage development and homeostasis

**DOI:** 10.1038/s41467-020-17630-6

**Published:** 2020-07-30

**Authors:** Yi Yao, Queping Liu, Indra Adrianto, Xiaojun Wu, James Glassbrook, Namir Khalasawi, Congcong Yin, Qijun Yi, Zheng Dong, Frederic Geissmann, Li Zhou, Qing-Sheng Mi

**Affiliations:** 1 Center for Cutaneous Biology and Immunology Research, Department of Dermatology, Henry Ford Health System, Detroit, MI 48202 USA; 2 Immunology Research Program, Henry Ford Cancer Institute, Henry Ford Health System, Detroit, MI 48202 USA; 3 Department of Public Health Sciences, Henry Ford Health System, Detroit, MI 48202 USA; 4 Center for Bioinformatics, Henry Ford Health System, Detroit, MI 48202 USA; 5 Department of Biochemistry, Microbiology, and Immunology, School of Medicine, Wayne State University, Detroit, MI 48202 USA; 6 Department of Cellular Biology and Anatomy, Augusta University, Augusta, GA 30912 USA; 7 Immunology Program, Sloan Kettering Institute, Memorial Sloan Kettering Cancer Center, New York, NY 10065 USA; 8 Department of Internal Medicine, Henry Ford Health System, Detroit, MI 48202 USA; 9 Present Address: Department of Pathology, Xiangya Hospital of Central South University, Changsha, Hunan 410008 China

**Keywords:** Epigenetics in immune cells, Monocytes and macrophages, Alveolar macrophages, Epigenetics

## Abstract

Alveolar macrophages (AMs) derived from embryonic precursors seed the lung before birth and self-maintain locally throughout adulthood, but are regenerated by bone marrow (BM) under stress conditions. However, the regulation of AM development and maintenance remains poorly understood. Here, we show that histone deacetylase 3 (HDAC3) is a key epigenetic factor required for AM embryonic development, postnatal homeostasis, maturation, and regeneration from BM. Loss of HDAC3 in early embryonic development affects AM development starting at E14.5, while loss of HDAC3 after birth affects AM homeostasis and maturation. Single-cell RNA sequencing analyses reveal four distinct AM sub-clusters and a dysregulated cluster-specific pathway in the HDAC3-deficient AMs. Moreover, HDAC3-deficient AMs exhibit severe mitochondrial oxidative dysfunction and deteriorative cell death. Mechanistically, HDAC3 directly binds to *Pparg* enhancers, and HDAC3 deficiency impairs *Pparg* expression and its signaling pathway. Our findings identify HDAC3 as a key epigenetic regulator of lung AM development and homeostasis.

## Abstract (web summary)

Alveolar macrophages are known to derive from embryonic precursors although the regulation of this process is poorly understood. Here the authors propose a key role for histone deacetylase 3 as an epigenetic regulator of lung alveolar macrophage development.

## Introduction

Alveolar macrophages (AMs), the resident macrophages in lung alveoli, are important for the maintenance of homeostasis in the airways and are involved in the development of a variety of pulmonary diseases^1^. Recent lineage-tracing studies have demonstrated that tissue-resident macrophages (TRMs) originate from embryonic yolk sac (YS) erythro-myeloid progenitors (EMPs) and can self-renew in most adult tissues at steady state without contribution from bone marrow (BM) hematopoietic stem cells (HSCs)^2–4^. YS EMP cells differentiate into premacrophages (pMacs) that colonize all embryonic organs as TRMs starting at embryonic day 9.5 (E9.5)^5^. The YS EMPs also enter the fetal liver (FL) starting from E12.5, where they expand and differentiate into pMacs and/or monocytes that travel to local tissues through the blood and eventually become tissue-specific TRMs^4^. Fetal monocytes begin to accumulate in the developing lung at E14.5 and then differentiate into immature AMs (preAMs) expressing F4/80^int^CD11b^int^, which postnatally mature into AMs expressing CD11c^hi^Siglec-F^hi^CD11b^lo,^^4,6,7^. Like the majority of other TRMs, AMs self-maintain locally at steady state throughout adult life^4,8^. Under stressed conditions, AMs are either self-maintained by local proliferation or completely replaced by bone marrow-derived cells in a challenge-dependent manner^9,10^.

A few genes have been identified as requirements for AM development and homeostasis. Granulocyte macrophage colony-stimulating factor (GM-CSF) was shown to be essential for the perinatal differentiation of fetal monocytes into preAMs and for the full maturation of AMs postnatally^7^. Transforming growth factor-β (TGF-β) is also crucial for the embryonic differentiation of fetal monocytes into preAMs, their postnatal maturation, as well as the homeostasis of adult AMs^11^. Both GM-CSF and TGF-β signaling pathways induce the expression of PPAR-γ, a signature transcription factor essential for the development of AMs^7,11^. Macrophage identity and homeostasis require the precise regulation of gene expression that is governed by different epigenetic mechanisms, such as DNA methylation, histone modification, and chromatin structure^12^. However, very little is known about the epigenetic factors that regulate AM development from EMPs and fetal monocytes, as well as its postnatal homeostasis.

Histone deacetylases (HDACs) are enzymes that regulate gene expression by modifying chromatin structure through the removal of acetyl groups from target histones or through direct deacetylation of non-histone proteins. HDACs exhibit limited substrate specificity and rely on transcription factors with specific DNA-binding and/or chromatin-binding activities in order to target specific genes^13^. Class I HDACs (HDAC1, 2, 3, 8) can assemble into multi-component co-repressor^13^ or co-activator^14^ complexes that regulate the transcription of a broad array of genes which fundamentally impact cellular physiology, organism development, and disease pathogenesis^15–18^. Several pan HDAC inhibitors (HDACi) have been approved by the FDA for anti-tumor therapy^19^. More recently, HDACi was suggested for use in the treatment of asthma and other inflammatory lung diseases^20,21^. HDAC3, belonging to the class I HDAC family, is required for the macrophage inflammatory gene expression program^22^ and serves as an epigenetic brake in macrophage alternative activation^23^. However, it remains unknown whether HDAC3 is involved in the ontogeny and homeostasis of AMs.

In this study, we aim to investigate the role of HDAC3 in the embryonic development and postnatal maintenance of AMs. Using mice with TRM or AM lineage-specific or time-specific HDAC3 deletion and the bone marrow chimeric mouse model, we demonstrate that HDAC3 deficiency leads to severe impairment of AM ontogeny, maintenance, maturation, and regeneration. Single-cell RNA sequencing analyses reveal that AMs have four unique sub-populations and that a lack of HDAC3 gives rise to AM subset-specific pathway dysregulation. Moreover, loss of HDAC3 results in dysregulated mitochondrial oxidative phosphorylation and cell survival. Mechanistically, HDAC3 directly binds to *Pparg* gene enhancers and regulates PPAR-γ signaling during AM development. Our findings uncover HDAC3 as a key epigenetic factor in the regulation of lung AM embryonic development and maintenance after birth.

## Results

### HDAC3 is required for the embryonic development of AMs

**Fig. 1:**
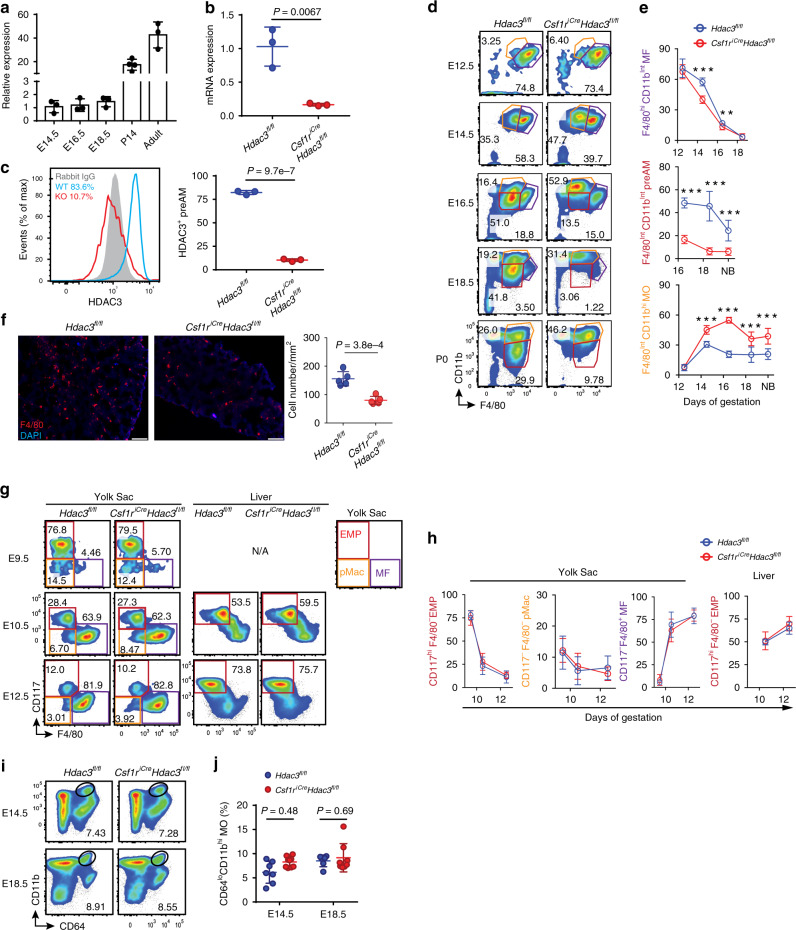
HDAC3 is required for embryonic development of AMs. **a** qRT-PCR analysis of HDAC3 mRNA expression in lung MFs, preAMs, or AMs from C57BL/6 mice (*n* = 4 for postnatal day 14 (P14), *n* = 3 for each other time point). The expression of mRNA (**b**) and protein (**c**) of HDAC3 in preAMs determined by qRT-PCR and flow cytometry, respectively, from *n* = 3 *Hdac3*^*fl/fl*^;*Csf1r*^*iCre*^*Hdac3*^*fl/fl*^ newborns (P0-P1). Frequencies of HDAC3-expressing (HDAC3^+^) AMs are shown in **c**. **d** Representative flow cytometry plots: gated from CD45^+^ live cells of fetal lung. **e** Frequencies of lung MFs, preAMs, and MOs as in **d**. E12.5: *n* = 14 *Hdac3*^*fl/fl*^, *n* = 5 *Csf1r*^*iCre*^*Hdac3*^*fl/fl*^; E14.5: *n* = 5 *Hdac3*^*fl/fl*^, *n* = 11 *Csf1r*^*iCre*^*Hdac3*^*fl/fl*^, ***P = 6.3e−7 (MF), 9.0e−6 (MO); E16.5: *n* = 12 *Hdac3*^*fl/fl*^, *n* = 9 *Csf1r*^*iCre*^*Hdac3*^*fl/fl*^, **P = 7.1e−3 (MF), ***P = 2.1e−13 (preAM), 5.3e−16 (MO); E18.5: *n* = 14 *Hdac3*^*fl/fl*^, *n* = 19 *Csf1r*^*iCre*^*Hdac3*^*fl/fl*^, ***P = 5.7e–14 (preAM), 6.5e−7 (MO); newborns (NB): *n* = 12 *Hdac3*^*fl/fl*^, *n* = 6 *Csf1r*^*iCre*^*Hdac3*^*fl/fl*^, ***P = 2.0e−4 (preAM), 2.9e−5 (MO). **f** Lung tissue sections from E18.5 embryos as in **d**. Scale bars, 100 μm. Cell numbers (*n* = 5 *Hdac3*^*fl/fl*^;*Csf1r*^*iCre*^*Hdac3*^*fl/fl*^) are shown per tissue area (mm^2^). **g** Representative flow cytometry plots: gated from CD45^+^Lin^‒^ (lineage negative) cells in YS and CD45^+^ live cells in fetal liver. **h** Frequencies of EMPs, pMacs, and MFs as in **g**. E9.5: *n* = 13 *Hdac3*^*fl/fl*^, *n* = 11 *Csf1r*^*iCre*^*Hdac3*^*fl/fl*^; E10.5: *n* = 6 *Hdac3*^*fl/fl*^, *n* = 10 *Csf1r*^*iCre*^*Hdac3*^*fl/fl*^; E12.5: *n* = 8 *Hdac3*^*fl/fl*^, *n* = 5 *Csf1r*^*iCre*^*Hdac3*^*fl/fl*^. **i** Representative flow cytometry plots: gated from CD45^+^ live cells in fetal liver. **j** Frequencies of MO as in **i**. E14.5: *n* = 8 *Hdac3*^*fl/fl*^, *n* = 15 *Csf1r*^*iCre*^*Hdac3*^*fl/fl*^; E18.5: *n* = 6 *Hdac3*^*fl/fl*^, *n* = 8 *Csf1r*^*iCre*^*Hdac3*^*fl/fl*^. Each dot represents one embryo or newborn and bars represent mean ± SD of biologically-independent samples in each panel. All *P* values obtained by Student’s two-tailed unpaired *t* test. MF fetal macrophages, MO monocytes. Source data are provided as a Source Data file.

Before birth, F4/80^hi^CD11b^int^ pMac-derived fetal macrophages^5^ and F4/80^int^CD11b^hi^ FL monocytes sequentially colonize the developing lung around E12.5 and E14.5, respectively^8^. Fetal monocytes further differentiate into F4/80^int^CD11b^int^ preAMs, which become CD11c^hi^Siglec-F^hi^CD11b^lo^ AMs during the first week of life. To investigate the role of HDAC3 in AMs, we first examined the expression pattern of HDAC3 in the lung fetal macrophages and preAMs during embryonic development, as well as AMs at a young age and adulthood, using qRT-PCR. As shown in Fig. 1a, HDAC3 was expressed in lung fetal macrophages at E14.5 and in preAMs at E16.5 and E18.5, but its expression was dramatically increased in AMs from the young (P14, ~20-fold) and adult (~40-fold) mice. These results suggest that HDAC3 is potentially involved in AM embryonic development and maintenance after birth.

To determine whether HDAC3 regulates embryonic development of AMs, we generated *Csf1r*^*iCre*^*Hdac3*^*fl/fl*^ conditional knockout (cKO) mice, in which HDAC3 is deficient in the *Csf1r*-expressing myeloid cells, including TRMs and monocytes^24,25^. Both HDAC3 mRNA and protein were efficiently depleted (>80%) in preAMs from the HDAC3cKO newborns as determined by qRT-PCR and flow cytometry, respectively (Fig. 1b, c). We next examined the fetal macrophages, preAMs, and fetal monocytes in the lung from *Csf1r*^*iCre*^*Hdac3*^*fl/fl*^ cKO mouse embryos and wild type (WT) littermates between E12.5 and right after birth (P0). There was no significant alteration in the frequencies of F4/80^hi^CD11b^lo^ fetal macrophages and F4/80^lo^CD11b^hi^ monocytes in the lung between cKO and WT embryos at E12.5 (Fig. 1d, e, gates shown in Supplementary Fig. 1a). However, a modest but significant reduction in the frequency of fetal macrophages was observed in HDAC3cKO embryos at E14.5 and E16.5, followed by a gradual diminish afterwards. On the other hand, the frequency of F4/80^int^CD11b^int^ preAM cells that emerged at E16.5 in the fetal lung was dramatically decreased in HDAC3cKO compared to WT littermates starting at E16.5 until birth. In contrast, fetal monocytes showed compensatorily increased distribution in the absence of HDAC3 starting at E14.5. Immunofluorescence staining of lung tissue sections at E18.5 further confirmed that the number of lung preAMs was significantly reduced in the HDAC3cKO mice (Fig. 1f). Collectively, these results suggest that HDAC3 is indispensable for the development of YS pMac-derived lung macrophages and FL monocyte-derived preAMs during embryogenesis.

Next, we investigated whether HDAC3 deficiency blocks EMP development and differentiation into pMacs and FL monocytes in the YS and FL, respectively. Interestingly, within the CD45^+^Lin^–^ (CD45-expressing; lineage negative) population, the frequencies of CD117^hi^F4/80^–^ EMPs, CD117^–^F4/80^–^ pMacs, and CD117^–^F4/80^+^ macrophages in the YS remained unaltered in HDAC3cKO embryos compared to the WT at E9.5, E10.5, and E12.5 (Fig. 1g, h, gates shown in Supplementary Fig. 1b), suggesting that HDAC3 is dispensable for EMPs and their further differentiation into pMacs and YS macrophages. Furthermore, we observed comparable frequencies of EMPs in the FL between the HDAC3cKO and WT embryos at E10.5 and E12.5, suggesting that the loss of HDAC3 also does not affect EMP seeding in the FL (Fig. 1g, h). In addition, the frequency of CD64^lo^CD11b^hi^Ly6c^hi^ FL monocytes remained unaltered during the embryonic stage (E14.5-E18.5) in the HDAC3cKO mice (Fig. 1i, j, gates shown in Supplementary Fig. 1c). HDAC3 deletion in FL monocytes, which was confirmed by qRT-PCR and flow cytometry (Supplementary Fig. 2a, b), does not disrupt their differentiation into TRMs in other organs, such as the brain, spleen, kidney, and pancreas (Supplementary Fig. 3a, b, gates shown in Supplementary Fig. 1a). Overall, HDAC3 deficiency does not affect EMPs, their ability to differentiate into pMacs in the YS, or FL monocyte development, but it may play key roles in pMac-derived macrophages and FL monocyte-derived preAMs in the lung.

### HDAC3 is essential for the maintenance of AMs after birth

**Fig. 2:**
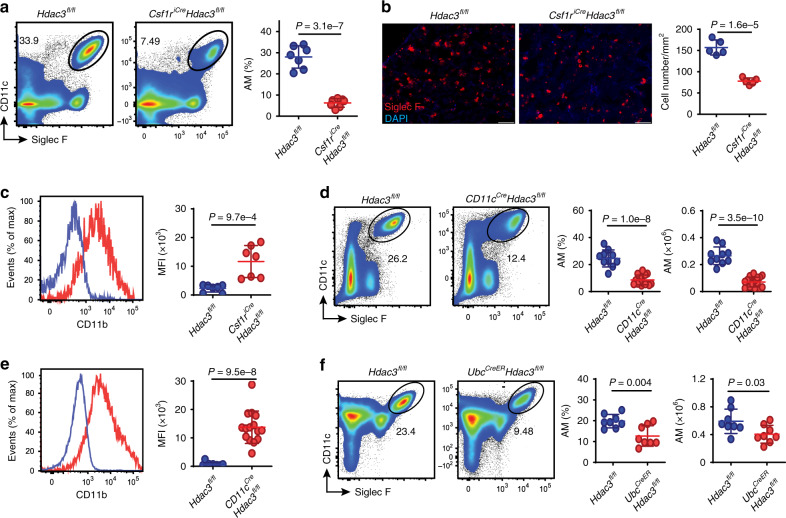
HDAC3 is essential for the maintenance of mature AMs in the adult mice. **a** Flow cytometry for CD11c and Siglec-F expression of lung AMs within CD45^+^ live cells from *n* = 7 *Hdac3*^*fl/fl*^; *Csf1r*^*iCre*^*Hdac3*^*fl/fl*^ adult mice. Frequencies of AMs are shown on the right. **b** Lung tissue sections from adult mice as in **a**. Scale bars, 100 μm. Cell numbers (*n* = 5 *Hdac3*^*fl/fl*^*;Csf1r*^*iCre*^*Hdac3*^*fl/fl*^) are shown per tissue area (mm^2^). **c** Histogram plot for CD11b expression of lung AMs from *n* = 7 *Hdac3*^*fl/fl*^;*Csf1r*^*iCre*^*Hdac3*^*fl/fl*^ adult mice. MFI, mean fluorescence intensity. **d** Flow cytometry analysis of AMs from *n* = 10 *Hdac3*^*fl/fl*^, *n* = 17 *Cd11c*^*Cre*^*Hdac3*^*fl/fl*^ adult mice. Frequencies and absolute numbers of AMs are shown on the right. **e** Histogram plot for CD11b expression of lung AMs from *n* = 11 *Hdac3*^*fl/fl*^, *n* = 15 *Cd11c*^*cre*^*Hdac3*^*fl/fl*^ adult mice. MFI of CD11b is shown on the right. **f** Flow cytometry analysis of AMs from *n* = 8 *Hdac3*^*fl/fl*^*;Ubc*^*CreER*^*Hdac3*^*fl/fl*^ adult mice. Frequencies and absolute numbers of AMs are shown on the right. Each dot represents one mouse and bars represent mean ± SD of biologically-independent samples. All *P* values were obtained using the Student’s two-tailed unpaired *t* test. Source data are provided as a Source Data file.

We next examined the AMs from the *Csf1r*^*iCre*^*Hdac3*^*fl/fl*^ cKO adult mice. As expected, the frequency of lung AMs (CD11c^hi^Siglec-F^hi^) was markedly decreased in the HDAC3cKO adult mice (Fig. 2a, gates shown in Supplementary Fig. 1d). This reduction was further confirmed by immunofluorescence staining of frozen tissue sections (Fig. 2b). After birth, preAMs quickly downregulate their CD11b expression and become mature AMs^7^. As shown in Fig. 2c, the remaining AMs in the HDAC3-deficient mice were found to highly express CD11b. This suggests that maintenance of mature AMs in the adult mice is very likely dependent on HDAC3. However, given that Csf1r.Cre-induced gene deletion occurs in the embryonic stage, the AM defects observed in the *Csf1r*^*iCre*^*Hdac3*^*fl/fl*^ cKO adult mice may be due to embryonic developmental dysregulation rather than a maintenance abnormality after birth.

To address this question, we next generated mice with a constitutive HDAC3 deficiency in AMs postnatally by crossing *Hdac3*^*fl/fl*^ mice with *CD11c*^*Cre*^ transgenic mice, given that CD11c is highly expressed in AMs after birth^7^. We found that the frequency and number of AMs were dramatically reduced in *CD11c*^*Cre*^*Hdac3*^*fl/fl*^cKO mice compared to their WT counterparts (Fig. 2d), and that CD11b was highly expressed on AMs from *CD11c*^*Cre*^*Hdac3*^*fl/fl*^cKO mice (Fig. 2e). We further generated inducible HDAC3-deficient mice by crossing *Hdac3*^*fl/fl*^ mice with ubiquitin C promoter-driven, tamoxifen-inducible Cre (*Ubc*^*CreER*^) transgenic mice, in which tamoxifen (TAM) timely induces HDAC3 deletion. *Ubc*^*CreER*^*Hdac3*^*fl/fl*^ mice (6–8 weeks old) were treated with TAM for 5 consecutive days, then lung AMs were analyzed 1 week and 3–5 weeks after TAM treatment. Successful HDAC3 deletion in the targeted cells was confirmed by qRT-PCR (Supplementary Fig. 4a). Although normal distribution of AMs was observed in *Ubc*^*CreER*^*Hdac3*^*fl/fl*^ mice at 1 week post-TAM treatment (Supplementary Fig. 4b), lack of HDAC3 led to a significant reduction in the frequency and number of lung AMs at 3–5 weeks post-treatment (Fig. 2f). Taken together, these findings suggest that HDAC3 also plays a key role in the maintenance of AM homeostasis, as well as their maturation after birth at steady state.

### Regeneration of AMs from the BM depends on HDAC3

**Fig. 3:**
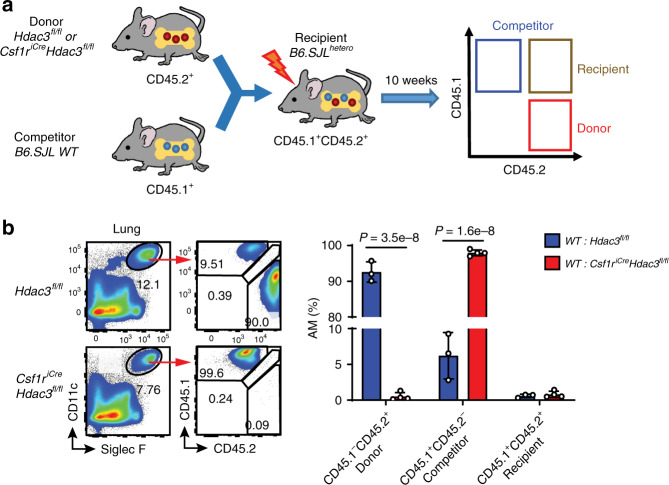
Regeneration of AMs from bone marrow depends on HDAC3. The bone marrow (BM) cells from *Hdac3*^*fl/fl*^ and *Csf1r*^*icre*^*Hdac3*^*fl/fl*^ mice (CD45.2^+^) were co-transferred with competitor BM cells from B6.SJL mice (CD45.1^+^) into lethally-irradiated B6.SJL^hetero^ mice (CD45.1^+^CD45.2^+^). The lungs were harvested from the recipient mice 10 wks after reconstitution. **a** Schematic representation of the BM chimeric mouse working models. **b** Flow cytometry of CD45.1 and CD45.2 expression on CD11c^hi^Siglec-F^hi^ lung AMs from *n* = 3 *WT: Hdac3*^*fl/fl*^, *n* = 4 WT: *Csf1r*^*icre*^*Hdac3*^*fl/fl*^ mice. Bars represent mean ± SD of biologically-independent samples. All *P* values were obtained using the Student’s two-tailed unpaired *t* test. Source data are provided as a Source Data file.

AMs self-maintain at steady state independently of circulating precursors^9^. However, under certain inflammatory conditions or BM transplantation following lethal whole-body irradiation, BM-derived monocytes can repopulate the AM niche^9,26^. We next sought to determine whether BM-derived AMs are also dependent on HDAC3. To do this, we established a BM chimeric mouse model by co-transferring BM cells from *Hdac3*^*fl/fl*^ or *Csf1r*^*iCre*^*Hdac3*^*fl/fl*^ cKO donors (CD45.2^+^) with competitor BM cells from B6.SJL WT mice (CD45.1^+^) into the irradiated B6.SJL^hetero^ (CD45.1^+^CD45.2^+^) recipient mice (Fig. 3a). As expected, in the chimeric mice, lung AMs were radiosensitive and largely replaced by BM-derived cells from both WT donor and competitor mice (Fig. 3b). However, the BM from HDAC3cKO mice completely failed to reconstitute lung AMs compared to the BM from WT mice. These findings suggest that HDAC3 is not only important for embryonically-derived AMs, but also required for the regeneration of AMs from adult BM under stress conditions.

### HDAC3 regulates AM subset-specific gene expression profile

**Fig. 4:**
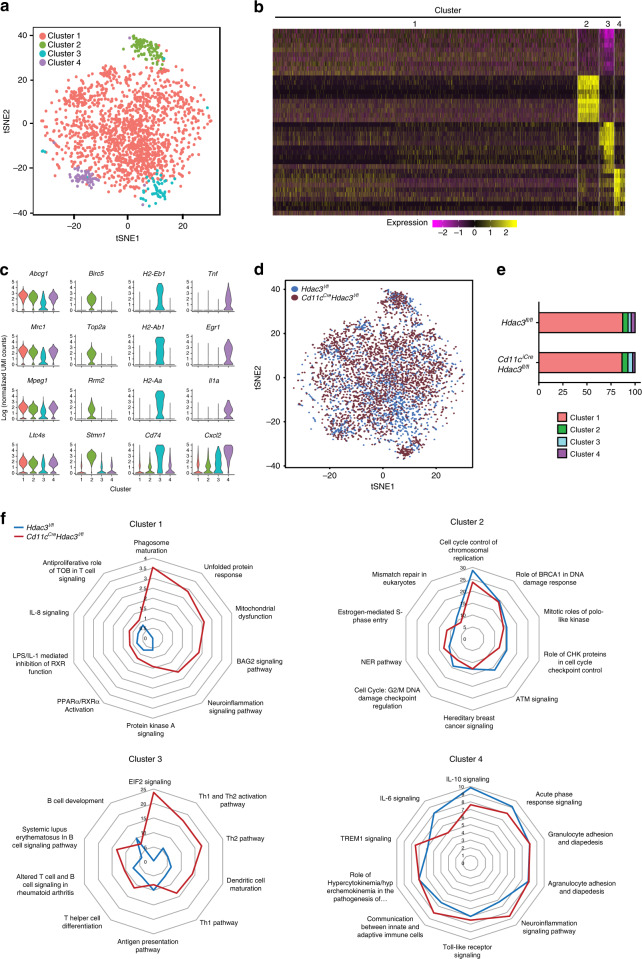
HDAC3 regulates AM subset-specific gene expression profiles. CD11c^hi^Siglec-F^hi^ lung AMs were sorted from *n* = 1 *Hdac3*^*fl/fl*^;*Cd11c*^*Cre*^*Hdac3*^*fl/fl*^ adult mouse for scRNA-seq analysis. **a** t-SNE map of individual cells clustered from 2055 *Hdac3*^*fl/fl*^ AM cells. **b** Heat map representing signature genes which were most highly expressed (top 10) in each AM cluster. **c** Representative violin plots showing signature genes expressed by individual AM subsets. **d** t-SNE maps of individual cells from the *Hdac3*^*fl/fl*^ and *Cd11c*^*Cre*^*Hdac3*^*fl/fl*^ (3067) AM cells. **e** The percentile of each AM subset within total AM population between two groups. **f** Radar maps depicting dysregulated pathways between two groups in each AM subset. Source data are provided as a Source Data file.

Recent single-cell studies have shown that heart and liver TRMs are heterogeneous populations at steady state^27,28^. To characterize whether lung AMs are also heterogeneous, we sorted CD11c^hi^Siglec-F^hi^ AMs from *Hdac3*^*fl/fl*^ WT adult mice and performed single-cell RNA-sequencing (scRNA-seq) using the 10× Genomics platform. More than 2000 cells were analyzed and T-distributed stochastic neighbor embedding (t-SNE) dimensionality reduction analysis identified 4 major AM clusters (Fig. 4a). Each AM sub-cluster has a unique gene expression pattern (Fig. 4b) with their signature genes (Fig. 4c). Of the AM clusters, cluster 1 was the most abundant and expressed macrophage-associated genes, such as *Abcg1, Mrc1*, and *Mpeg1*. Cluster 2 expressed genes such as *Birc5, Top2a, Rrm2*, and *Stmn1*, which are involved in the cell cycle and mitosis. Cluster 3 had relatively higher expression of antigen-presentation genes, such as *H2-Eb1, H2-Ab1, H2-Aa,* and *Cd74*. Cluster 4 was enriched in many genes associated with immune response, such as *Tnf, Il1a, Cxcl1, Cxcl2*, and *Cxcl3*. The cluster-specific gene expression pattern suggests that each AM cell subset may have a distinct role in AM maintenance and function.

In order to determine whether HDAC3 is required for the maintenance of a specific lung AM cell subset, we next performed scRNA-seq on the sorted AM cells from *Cd11c*^*Cre*^*Hdac3*^*fl/fl*^cKO mice and compared the AM clusters between HDAC3cKO and WT mice. Although the composition of clusters 1 and 2 remained almost unaltered in the KO, the frequency of cluster 3 was increased from 3.3% to 4.9%, while the frequency of cluster 4 was reduced from 4.0% to 2.4% in the absence of HDAC3 (Fig. 4d, e). This suggests that the loss of HDAC3 may affect the maintenance of AM subsets in a cluster-specific manner. In addition, we also observed certain cluster-specific transcriptomic changes between WT and cKO mice (Supplementary Fig. 5). Ingenuity pathway analysis (IPA) revealed that each AM cluster in the cKO mice exhibited distinct dysregulated pathways compared to that in their WT counterpart (Fig. 4f). In cluster 1, the key disordered pathways included phagosome maturation, unfolded protein response, mitochondria dysfunction, and BAG2 signaling pathways, which are associated with macrophage phagocytosis, polarization, survival, and stress responses. In cluster 2, the pathways related to cell cycle control were less enriched in the cKO cells. In cluster 3, several immune-responsive pathways were upregulated in the cKO cells, including E1F2 signaling, Th1/Th2 pathways, and dendritic cell maturation. In cluster 4, the pathways involved in cytokine signaling (i.e., IL-6 and IL-10 signaling) and acute phase response signaling were less enriched in the cKO cells, whereas Toll-like receptor signaling, neuroinflammation signaling pathway, and communication between innate and adaptive immune cells, were strongly enriched in the cKO cells. These data reveal that HDAC3 deficiency results in cluster-specific pathway dysregulation in AMs at steady state, which may lead to distinct function disorders in each AM cluster.

Interestingly, when we analyzed transcriptomes of AM populations without clustering, we identified a total of 186 differentially expressed genes, including 59 upregulated genes and 127 downregulated genes in the HDAC3-deficient AMs compared to their WT counterparts (Supplementary Fig. 6a). IPA of these differentially expressed genes showed that several canonical pathways related to inflammation and immune responses were significantly inhibited in the HDAC3cKO AMs (Supplementary Fig. 6b). Moreover, the signaling pathways related to macrophage survival and apoptosis were also remarkably impaired, including TREM1 signaling^29,30^, NF-kB pathway^31,32^, and STAT3 pathway^33,34^. qRT-PCR analysis further confirmed the downregulation of the genes associated with these three pathways, such as *Flt1, Mapk3, Map3k1, Tlr2, Fcgr2b, Tgfb1*, and *Tgfbr1* (Supplementary Fig. 6c). These data suggest that the inhibition of TREM1, NF-kB, and STAT3 signaling pathways might reduce cell survival of HDAC3cKO AMs, leading to the decreased total number of AM cells in the absence of HDAC3 as we observed at steady-state maintenance (Fig. 2d). On the other hand, scRNA-seq also revealed that PPAR-γ was remarkably reduced in HDAC3-deficient AMs (Supplementary Fig. 6a), suggesting that HDAC3 might control AM homeostasis through regulation of PPAR-γ.

### HDAC3 controls mitochondria function and survival of preAMs

**Fig. 5:**
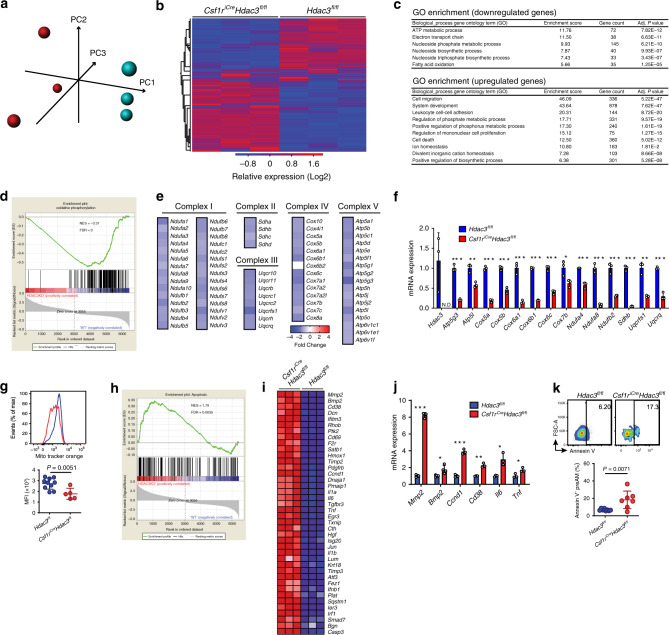
HDAC3 controls metabolism and cell death during AM embryonic development. Bulk RNA-seq analysis of sorted preAMs from fetal lung of *n* = 3 *Hdac3*^*fl/fl*^;*Csf1r*^*iCre*^*Hdac3*^*fl/fl*^ embryos at E18.5. **a** Principle component analysis (PCA) performed on transcriptome (*Hdac3*^*fl/fl*^, turquoise; *Csf1r*^*iCre*^*Hdac3*^*fl/fl*^, red). **b** Heat map showing differential gene expression (*P* < 0.05). **c** Gene Ontology (GO) enrichment of the downregulated and upregulated genes in **b**. The biological processes with the smallest adjusted *P* values by EASE Score (one-tailed Fisher’s exact *P*-value in DAVID system) with the Benjamini-Hochberg correction for multiple testing from each class are shown. **d** Enrichment plot of oxidative phosphorylation from Gene Set Enrichment Analysis (GSEA) of differentially expressed genes. NES, Normalized Enrichment Score. **e** Heat map depicting downregulated genes related to oxidative phosphorylation identified by GSEA. **f** qRT-PCR analysis of mRNA expression levels of oxidative phosphorylation-associated genes. *n* = 3 *Hdac3*^*fl/fl*^;*Csf1r*^*iCre*^*Hdac3*^*fl/fl*^. *P = 0.03 (*Cox7b*); **P = 4.2e−3 (*Atp5l*), 1.2e–3 (*Uqcrfs1*); ***P = 1.0e–4 (*Atp5g3*), 2.7e−5 (*Cox5a*), 4.7e−4 (*Cox5b*), 5.7e−4 (*Cox6a1*), 1.2e−6 (*Cox6b1*), 2.4e−4 (*Cox6c*), 7.3e−4 (*Ndufa4*), 1.6e−4 (*Ndufa8*), 8.0e–4 (*Ndufb2*), 1.5e–5 (*Sdhb*), 2.2e−4 (*Uqcrq*). **g** Histogram plot for MitoTracker Orange dye (mitochondria membrane potential) staining of preAMs determined by flow cytometry. MFI median fluorescence intensity; *n* = 10 *Hdac3*^*fl/fl*^, *n* = 5 *Csf1r*^*iCre*^*Hdac3*^*fl/fl*^. **h** GSEA plot of apoptosis. **i** Heat map showing upregulated genes related to apoptosis identified by GSEA. Colors (red to blue) show expression values (high to low). **j** qPCR analysis of mRNA expression levels of apoptosis-associated genes. BMP2: *n* = 5 *Hdac3*^*fl/fl*^, *n* = 4 *Csf1r*^*iCre*^*Hdac3*^*fl/fl*^; other genes: *n* = 3 *Hdac3*^*fl/fl*^;*Csf1r*^*iCre*^*Hdac3*^*fl/fl*^. *P = 0.01 (*Bmp2*), 0.011 (*Il6*), 0.026 (*Tnf*); **P = 1.1e–3 (*Cd38*); ***P = 4.7e−6 (*Mmp2*), 1.3e−4 (*Ccnd1*). **k** Flow cytometry of annexing V staining of preAMs; *n* = 8 *Hdac3*^*fl/fl*^, *n* = 7 *Csf1r*^*iCre*^*Hdac3*^*fl/fl*^. Each dot represents one embryo at E18.5 and bars represent mean ± SD of biologically-independent samples in each panel. All *P* values were obtained using the Student’s two-tailed unpaired *t* test. Source data are provided as a Source Data file.

To further obtain mechanistic insights into the embryonic deficiency of AMs in the absence of HDAC3, we first performed bulk RNA-seq analysis of lung preAMs from cKO and WT at E18.5. Based on the principal component analysis (PCA) of their transcriptomes, the molecular signatures showed a clear difference in preAMs between cKO and WT (Fig. 5a). A total of 9074 differentially expressed genes (*P* < 0.05) were identified in the preAMs between HDAC3cKO and WT, including 4612 downregulated and 4462 upregulated genes (Fig. 5b, Supplementary Data 1). Gene Ontology (GO) analysis further identified the altered biological pathways in the HDAC3cKO preAMs, including multiple metabolic pathways, the electron transport chain, cell proliferation, and cell death (Fig. 5c, Supplementary Data 2). Most of the identified pathways are consistent with previous studies in other cell types with HDAC3 deletion^14,35,36^.

Furthermore, gene set enrichment analysis (GSEA) identified some gene sets that were either depleted (normalized enrichment score, NES < 0, false discovery rate, FDR < 0.05) (Supplementary Fig. 7a, b) or enriched in HDAC3cKO phenotype (NES > 0, FDR < 0.05) (Supplementary Fig. 8a, b). Notably, the genes involved in oxidative phosphorylation were significantly downregulated in HDAC3-deficient preAMs (Fig. 5d). Many subunits of protein complexes I-V of the electron transport chain, the key component of the oxidative phosphorylation process, were diminished in the absence of HDAC3 (Fig. 5e, f). In addition, we observed a significant decrease in Mitotracker Orange fluorescence-labeled AMs from cKO, which reflects the reduced mitochondria membrane potential and indicates impaired mitochondria function in HDAC3-deficient lung preAMs (Fig. 5g). Moreover, GSEA demonstrated that the absence of HDAC3 led to a strong enrichment of genes involved in apoptosis signaling (Fig. 5h–j). Enhanced apoptosis of preAMs at E18.5 was further confirmed by flow cytometry (Fig. 5k). These results suggest that HDAC3 regulates preAM mitochondrial oxidative function and cell death, which may be related to defective embryonic AM development in HDAC3 cKO mice.

### HDAC3 deficiency causes impaired PPAR-γ signaling in preAMs

**Fig. 6:**
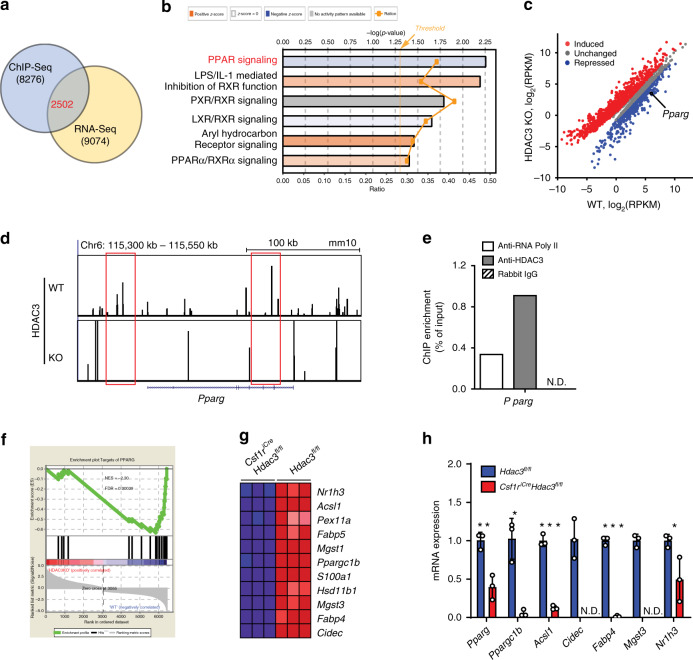
HDAC3 deficiency leads to impaired PPAR-γ signaling in AM development. **a** Venn diagram of genes identified by HDAC3 ChIP-seq in the AMs from *Hdac3*^*fl/fl*^ adult mice overlapped with differentially expressed genes from bulk RNA-seq. **b** Ingenuity Pathway Analysis (IPA) showing canonical nuclear receptor pathways of the overlapped genes as in **a**. Threshold indicates the minimum significance level by the right-tailed Fisher’s exact test. Ratio refers to the number of molecules from the dataset that map to the pathway listed divided by the total number of molecules that map to the canonical pathway from within the IPA knowledgebase. **c** Scatter plot of bulk RNA-seq data showing the overlapped genes as in **a** from *n* = 3 *Hdac3*^*fl/fl*^ WT; *Csf1r*^*iCre*^*Hdac3*^*fl/fl*^ KO littermates. Fold change >1.5 up (red) or down (blue) and false discovery rate (FDR) < 0.05. RPKM, reads per kilobase per million. **d** Genome browser tracks of the *Pparg* locus highlighting ChIP-seq data at the enhancers (boxed). *Hdac3*^*fl/fl*^, WT; *Csf1r*^*iCre*^*Hdac3*^*fl/fl*^, KO. **e** ChIP-qPCR data representing HDAC3 binding at the *Pparg* enhancers in MH-S cells. Enrichment value is normalized to input measurements. Representative data from two independent experiments. **f** GSEA plot of targets of PPAR-γ from bulk RNA-seq. **g** Heat map representing downregulated genes related to PPAR-γ signaling identified by GSEA as in **f**. Colors (red to blue) show expression values (high to low). **h** qPCR analysis of mRNA expression levels of the genes associated with PPAR-γ signaling. *n* = 3 *Hdac3*^*fl/fl*^;*Csf1r*^*iCre*^*Hdac3*^*fl/fl*^. *P = 0.043 (*Nr1h3*); **P = 5.5e−3 (*Pparg*), 3.4e−3 (*Ppargc1b*); ***P = 4.9e−5 (*Acsl1*), 6.4e–6 (*Fabp4*) by the Student’s two-tailed unpaired *t* test. Bars represent mean ± SD of biologically-independent samples in each panel. Source data are provided as a Source Data file.

We next sought to identify the direct downstream targets of HDAC3 for AM development. To achieve this, we performed HDAC3 ChIP-Seq analysis of lung AMs from the WT mice, which was further integrated with our bulk RNA-seq data. We found 2502 genes shared in both datasets that may be primary targets of HDAC3 transcriptional regulation (Fig. 6a). IPA analysis of these differentially expressed genes revealed that PPAR signaling was one of the canonical pathways potentially targeted by HDAC3 (Fig. 6b). Indeed, the repression of *Pparg* mRNA expression was correlated with the loss of HDAC3 in HDAC3-deficient preAMs (Fig. 6c). ChIP-seq analysis also showed that HDAC3 can directly bind to *Pparg* enhancers that are located in the upstream (within −30 kilobases (kb)) or intronic (+49 kb) regions from the transcription start sites in lung AMs from the WT mice, but not in the HDAC3cKO mice (Fig. 6d, Supplementary Data 3). The binding of HDAC3 to the *Pparg* enhancers was further confirmed by ChIP-qPCR using MH-S cells, a mouse AM cell line (Fig. 6e). Moreover, the GSEA of bulk RNA-seq data revealed that the absence of HDAC3 led to a significantly weaker enrichment of genes involved in PPAR-γ signaling compared to WT (Fig. 6f, g). Lack of HDAC3 resulted in the downregulation of PPAR-γ target genes, including *Ppargc1b*, *Acsl1, Cidec, Fabp4, Mgst3*, and *Nr1h3*, which was further confirmed by qRT-PCR (Fig. 6h). Collectively, these results indicate that HDAC3 directly targets PPAR-γ in AMs, and that loss of HDAC3 fundamentally abrogates the PPAR-γ signaling pathway during AM development.

### PPAR-γ regulates mitochondria function and survival of preAMs

**Fig. 7:**
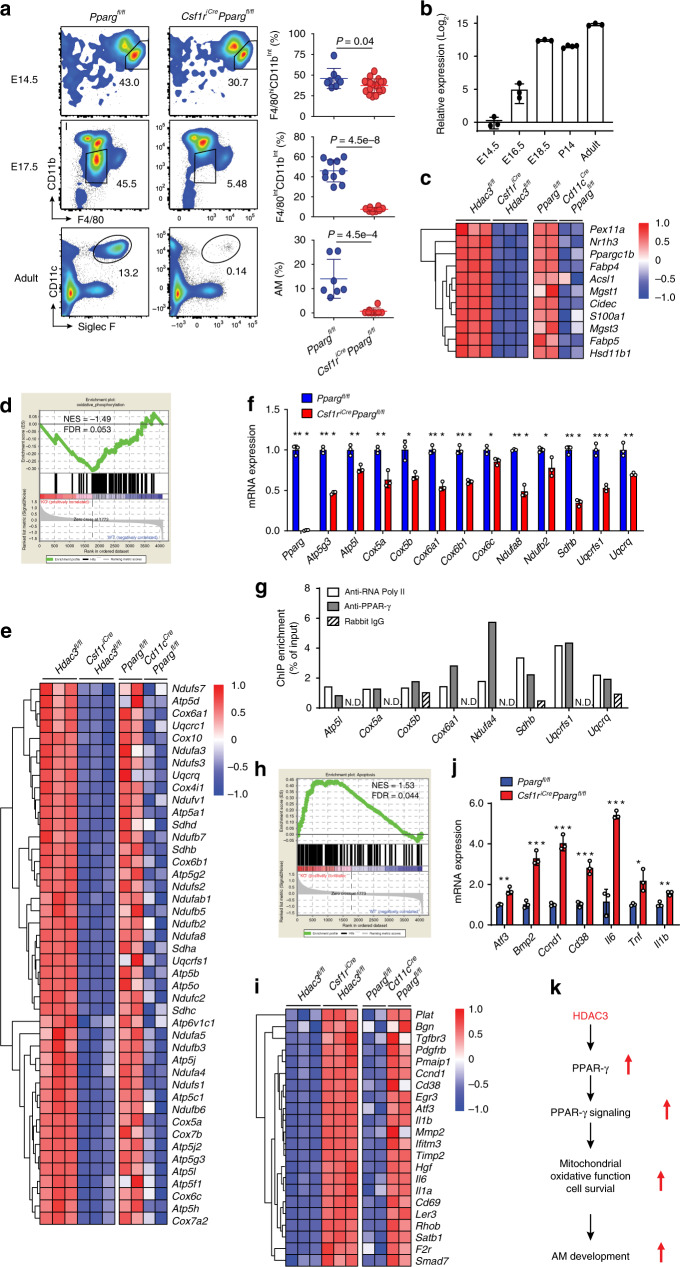
PPAR-γ governs mitochondria function and cell survival in AM development. **a** Flow cytometry analysis of lung fetal macrophages (F4/80^hi^CD11b^int^), preAMs (F4/80^int^CD11b^int^), and AMs (CD11c^hi^Siglec-F^hi^) from lungs of *Pparg*^*fl/fl*^ and *Csf1r*^*iCre*^*Pparg*^*fl/fl*^ mice at indicated ages. E14.5: *n* = 8 *Pparg*^*fl/fl*^, *n* = 19 *Csf1r*^*iCre*^*Pparg*^*fl/fl*^; E17.5: *n* = 10 *Pparg*^*fl/fl*^, *n* = 8 *Csf1r*^*iCre*^*Pparg*^*fl/fl*^; adult: *n* = 7 *Pparg*^*fl/fl*^, *n* = 8 *Csf1r*^*iCre*^*Pparg*^*fl/fl*^. **b** qRT-PCR analysis of PPAR-γ mRNA expression in the lung fetal macrophages (E14.5), preAMs (E16.5, E18.5), or AMs (P14, adult) isolated from C57BL/6 mice (*n* = 4 for P14, *n* = 3 for each other time point). Bulk RNA-seq data of preAMs from *n* = 3 *Hdac3*^*fl/fl*^;*Csf1r*^*iCre*^*Hdac3*^*fl/fl*^ embryos at E18.5 were generated as in Fig. 5. Microarray data for preAMs from *n* = 2 *Pparg*^*fl/fl*^;*Cd11c*^*Cre*^*Pparg*^*fl/fl*^ mice at day 2 after birth was obtained from GEO database (GSE60249). Heat map showing expression of genes associated with PPAR-γ signaling (**c**), oxidative phosphorylation (**e**), and apoptosis (**i**) from two datasets. GSEA plots presenting expression of genes associated with oxidative phosphorylation (**d**) and apoptosis (**h**). qRT-PCR analysis of mRNA expression levels of genes associated with oxidative phosphorylation (**f**) and apoptosis (**j**) in the preAMs isolated from *n* = 3 *Pparg*^*fl/fl*^;*Csf1r*^*iCre*^*Pparg*^*fl/fl*^ embryos at E17.5. *P = 0.012 (*Cox5b*), 0.019 (*Cox6c*), 0.035 (*Ndufb2*), 0.02 (*Tnf*); **P = 7.5e−3 (*Atp5l*), 4.4e−3 (*Cox5a*), 3.4e−3 (*Uqcrq*), 3.3e−3 (*Atf3*), 3.7e−3 (*Il1b*); ***P = 9.5e−6 (*Pparg*), 3.0e−5 (*Atp5g3*), 4.4e−4 (*Cox6a1*), 3.1e–4 (*Cox6b1*), 2.4e−4 (*Ndufa8*), 7.0e–5 (*Sdhb*), 6.5e−4 (*Uqcrfs1*), 5.2e−4 (*Bmp2*), 1.8e−4 (*Ccnd1*), 9.1e−4 (*Cd38*), 3.2e−4 (*Il6*). **g** ChIP-qPCR data representing PPAR-γ binding at the promoter regions of the indicated oxidative phosphorylation genes in MH-S cells. Enrichment value is normalized to input measurements. Representative data from two independent experiments. **k** Schema of HDAC3-PPAR-γ axis in regulation of AM development. Bars represent mean ± SD of biologically-independent samples in each panel. All *P* values were obtained using the Student’s two-tailed unpaired *t* test. Source data are provided as a Source Data file.

A prior study has shown impaired development of fetal lung preAMs in *Vav1-Cre-*induced, PPAR-γ-deficient mice at E18.5^7^, indicating that prenatal PPAR-γ is required for AM ontogeny. Using *Csf1r*^*iCre*^*Pparg*^*fl/fl*^cKO mice, we further found a modest reduction of F4/80^hi^CD11b^int^ YS pMac-derived lung fetal macrophages at E14.5 and a drastic loss of F4/80^int^CD11b^int^ fetal liver monocyte-derived preAMs at E17.5 from *Csf1r*^*iCre*^*Pparg*^*fl/fl*^cKO mice (Fig. 7a), which were consistent with the phenotype observed in HDAC3cKO mice (Fig. 1d, e). As expected, CD11c^hi^Siglec-F^hi^ AMs were essentially eliminated in the *Csf1r*^*iCre*^*Pparg*^*fl/fl*^cKO adult mice (Fig. 7a), which was consistent with the previous results using *Vav1*^*Cre*^- and *Cd11c*^*Cre*^*Pparg*^*fl/fl*^cKO mice^7^. Moreover, PPAR-γ gene expression was detected in the lung fetal macrophages at E14.5, which was gradually upregulated in preAMs at E16.5 and E18.5, and maintained at high levels in the AMs from young and adult mice (Fig. 7b). Interestingly, PPAR-γ mRNA was not expressed in the TRMs of the kidney, liver, brain, and heart (Supplementary Fig. 9), suggesting that HDAC3-PPAR-γ axis is specifically required for the development of lung AMs, but not for TRMs in other tissues.

We next revisited a recently-published microarray dataset^7^ and analyzed gene expression profiles of lung preAMs from *Pparg*^*fl/fl*^ WT and *Cd11c*^*Cre*^*Pparg*^*fl/fl*^cKO mice at day 2 after birth. As expected, the PPAR-γ signaling pathway in preAMs was severely impaired in the absence of PPAR-γ, which was consistent with our observations in *Csf1r*^*iCre*^*Hdac3*^*fl/fl*^cKO mice (Fig. 7c). Interestingly, oxidative phosphorylation-related genes, which were severely diminished in HDAC3cKO mice, were robustly reduced in PPAR-γ-deficient mice (Fig. 7d–f). ChIP-qPCR analysis using MH-S cells further showed an enrichment of PPAR-γ binding sites in the promoter regions (within 3 kb of the transcription start sites) of oxidative phosphorylation-related genes, including *Atp5l, Cox5a, Cox5b, Cox6a1, Ndufa4, Sdhb, Uqcrfs1*, and *Uqcrq*, all of which were downregulated in the absence of PPAR-γ (Fig. 7e, f), suggesting a possible direct impact of PPAR-γ on mitochondria function-related genes in AMs (Fig. 7g). Moreover, the apoptosis-associated genes were highly enriched in PPAR-γ-deficient mice compared to their WT counterparts, which was also consistent with our observations in HDAC3cKO mice (Fig. 7h–j). These data suggest that the PPAR-*γ* signaling pathway serves as a key pathway that contributes to the dysregulation of mitochondrial oxidative function and cell survival in the HDAC3-deficient AMs. Taken together, our study demonstrates that the HDAC3-PPAR-*γ* axis is indispensable for normal mitochondrial oxidative function and cell survival during AM development (Fig. 7k).

## Discussion

The concept of TRMs has been refreshed in the past few years. Recent lineage-tracing studies have revealed that adult mouse TRMs are derived from embryonic YS EMP-derived macrophages and FL monocytes rather than from bone marrow-derived precursors, and they are self-maintained at steady state after birth^2,24,37^. Although a few genes (e.g., *Csf2*, *Pparg*, and *Tgfb*) have been identified as requirements for AM development, little information is known about the epigenetic regulators that instruct the development and maintenance of AMs. HDAC3, belonging to the class I HDAC family, plays a critical role in many biological processes, including circadian rhythm^17^, intestinal homeostasis^38^, and tissue thermogenesis^14^. Here, we demonstrate that HDAC3 is required for embryonic development and postnatal maintenance of lung AMs at steady state. In addition, HDAC3 controls AM regeneration from BM progenitors after lethal whole-body irradiation. Our data further indicate that HDAC3 is dispensable for development of TRMs in the brain, spleen, kidney, and pancreas, suggesting that HDAC3 regulates TRM ontogeny in an organ-specific manner.

As the resident macrophages in lung alveoli, AMs are long lived with proliferative self-renewal capability, and control the maintenance of airway homeostasis through their capacity of clearing surfactant and cell debris at steady state^1,39^. During respiratory infection or inflammation, AMs also function as antigen-presenting cells by transporting exogenous antigens to lung-draining lymph nodes and releasing a wide array of cytokines and chemokines to reprogram both innate and adaptive immune responses^40,41^. However, it remains unclear whether multifunctional roles of AMs are derived from AM heterogeneity at the individual cell level. Recent advances in single-cell technology have provided a powerful tool to study tissue and cell heterogeneity^28,42–44^. To address if the AMs are heterogenous at steady state and if HDAC3 regulates AM heterogeneity and subset function, we performed scRNA-seq of mature AMs in the adult mice from WT and CD11cCre.HDAC3 KO mice. We found that the murine lung tissues contain four transcriptionally-distinct AM cell subsets. The most abundant cluster (cluster 1) accounted for over 85% of the total lung AMs and expressed high levels of genes related to lipid metabolism, including *Abcg1*^45^ and *Ltc4s*^46^. Thus, this abundant AM cluster may be specialized for pulmonary surfactant lipid clearance. It has been shown that the adult AM network is maintained throughout life by a slow local proliferation of lung AMs rather than from BM precursors^8^. Interestingly, one of the minor AM clusters (cluster 2, 6%) was characterized by higher expression of cell cycle-related genes, and may represent proliferating AM cells that slowly renew the AM network, which has yet to be further tested. Another minor AM cluster (cluster 3, 3%) was enriched in many antigen-presentation-related genes and may represent the AM subpopulation that possesses antigen-presenting function^47^. Furthermore, the most minor AM cluster (cluster 4, 4%) highly expressed a variety of genes involved in immune responses and may serve as the major source of AMs for secreting pro-inflammatory cytokines and chemokines during inflammatory conditions. Together, our data suggest that individual AM subpopulations might be specialized for unique functions to regulate lung homeostasis and antipathogen immunity.

Interestingly, HDAC3 deficiency resulted in unproportionable changes in the number of AM sub-cluster cells, indicating a cluster-dependent effect of HDAC3 on AM homeostasis. Moreover, the transcriptomic changes and their associated pathway dysregulation induced by HDAC3 deletion in AMs also showed a cluster-specific pattern, i.e., mitochondria dysfunction in cluster 1, cell cycle control disorders in cluster 2, antigen-presenting dysfunction in cluster 3, and immune response dysregulation in cluster 4. Notably, the mitochondria dysfunction found in the abundant AM cluster (cluster 1) is consistent with our observations from bulk-RNA seq data showing impaired mitochondrial oxidative function and deteriorative cell death in the absence of HDAC3 during AM development. Given the importance of mitochondria function in immune metabolism and cell survival of macrophages^48^, we reason that the loss of cluster 1 caused by impaired mitochondria function largely contributes to the reduced number of total AM cells in the HDAC3cKO mice. Moreover, the disorders of cell cycle control and DNA damage response in the proliferating cluster (cluster 2) likely lead to less proliferative capacity of this cluster, which may also partially contribute to the reduced number of HDAC3-deficient AM cells. On the other hand, loss of HDAC3 significantly affected the antigen-presenting function of cluster 3 and the immune-responsive function of cluster 4, suggesting that HDAC3 might crucially control the function of both AM clusters during immune responses. Further studies are needed to assess the role of HDAC3 in the functionality of individual AM subpopulations in the pathogenesis of lung diseases, such as asthma and cancer. Furthermore, it remains to be determined whether fetal immature preAMs have subpopulations and how HDAC3 regulates preAM subsets, if any, during embryonic development. However, given severe developmental defects on preAMs in the absence of HDAC3, we currently could not obtain enough cells from the KO littermates for scRNA-seq analysis. Advanced technology, e.g., mass cytometry, may serve as a promising and powerful tool to address these interesting questions in the future^49^.

Inhibition of HDACs is emerging as a promising approach to treat various types of malignant diseases, neurodegenerative diseases, inflammatory disorders, and cardiovascular diseases^50^. Four pan HDACi have been approved by the FDA as anticancer agents for treatment of lymphoma and multiple myeloma, while many other pan HDACi are currently in clinical trials for treatment of lymphoma and solid tumors^51^. HDACi have also been recently suggested in the treatment of asthma and other inflammatory lung diseases^20,21^. However, due to common side effects of these pan HDAC inhibitors, developing drugs with high selectivity for individual HDAC has drawn great attention. On the other hand, different subtypes of HDACs appear to play disparate roles in immune defense and disease progression^52^. Understanding the underlying mechanism by which individual HDACs regulate immune cell development, maintenance, and function will help us to identify potential therapeutic treatments. In the present study, deletion of HDAC3 leads to repressed PPAR-γ expression in fetal immature AMs, as well as adult mature AMs, as confirmed by bulk RNA-seq and scRNA-seq analyses, respectively, suggesting the essential role of the HDAC3-PPAR-γ axis in the development and maintenance of AMs. In addition, our data imply that the HDAC3-PPAR-γ axis controls fetal immature AM development, at least partially through regulation of mitochondria function and cell survival. Interestingly, PPAR-γ binding sites are enriched near the genes associated with oxidative phosphorylation, suggesting that PPAR-γ might directly regulate mitochondrial oxidative function in AMs. On the other hand, given lack of PPAR-γ expression in TRMs from other organs, the HDAC3-PPAR-γ axis seems to be only required for TRM development in a lung-specific manner. It should be noted that a very recent single-cell study revealed an enrichment of PPAR-γ^hi^ macrophages in human lung adenocarcinoma lesions that likely promotes an immunosuppressive microenvironment^53^. Thus, HDAC3i could be promising immunotherapeutic drugs to treat lung cancer patients by targeting the HDAC3-PPAR-γ axis in lung macrophage populations, which needs to be further investigated.

In conclusion, our data have provided evidence that HDAC3 serves as a key epigenetic regulator to control AM development at the embryonic stage, as well as in AM maintenance and regeneration after birth. Accumulated research has indicated that AMs are involved in the development of cancer, autoimmune, and inflammatory diseases in the airway. Thus, our findings may shed light on HDAC3 as a potential therapeutic target for AM-based intervention in cancer and autoimmune diseases.

## Methods

### Animals

*Hdac3*^*fl/fl*^ mice were provided by Scott W. Hiebert^54^. C57BL/6 (Strain #000664), B6.SJL (Strain #002014), *Cd11c*^*Cre*^ (Strain #008068), *Csf1r*^*iCre*^ (Strain #021024), *Ubc*^*CreER*^ (Strain #007001), and *Pparg*^*fl/fl*^ (Strain #004584) mice were purchased from the Jackson Laboratory (Bar Harbour, ME). To generate myeloid lineage-specific HDAC3 mutant mice, we crossed *Hdac3*^*fl/fl*^ and *Csf1r*^*iCre*^ mice (back to a B6/C57 mouse genetic background for 6 generations). To generate CD11c-expressing, cell-specific HDAC3 mutant mice, we crossed *Hdac3*^*fl/fl*^ and *Cd11c*^*Cre*^ mice. To generate inducible HDAC3 deletion mice, we crossed *Hdac3*^*fl/fl*^ and *Ubc*^*CreER*^ mice. To generate myeloid lineage-specific PPAR-γ-deficient mice, we crossed *Pparg*^*fl/fl*^ and *Csf1r*^*iCre*^ mice. To generate heterozygous mice expressing both CD45.1 and CD45.2, named B6.SJL^hetero^, we crossed B6.SJL (CD45.1) with C57BL/6 (CD45.2) mice.

Male and female mice from 6–18 weeks of age were used. All experiments included age-matched and sex-matched littermate controls. Embryonic development was estimated considering the day of vaginal plug formation as embryonic age of 0.5 days.

All mice were housed under specific pathogen-free conditions at temperatures of 20–26 °C with 30–70% humidity and a 12-h light/12-h dark cycle at Henry Ford Health System. Experimental animal protocols were performed in accordance with the guidelines of the Institutional Animal Care and Use Committee.

### Genotyping

Genotyping of *Csf1r*^*iCre*^^55^, *Cd11c*^*Cre*^^56^, *Ubc*^*CreER*^^57^, and *Pparg*^*fl/fl*^^58^ mice was performed in adherence to the standard PCR-based procedure. The following primers were used for each mouse strain: *Csf1r*^*iCre*^: forward 5′-CTAGGCCACAGAATTGAAAGATCT-3′ and reverse 5′-ATCAGCCACACCAGACACAGAGATC-3′; *Cd11c*^*Cre*^*:* forward 5′-ACTTGGCAGCTGTCTCCAAG-3′ and reverse 5′-GCGAACATCTTCAGGTTCTG-3′; *Ubc*^*CreER*^: forward 5′-GACGTCACCCGTTCTGTTG-3′ and reverse 5′-AGGCAAATTTTGGTGTACGG-3′; *Pparg*^*fl/fl*^: 5′-CTAGTGAAGTATACTATACTCTGTGCAGCC-3′ and 5′-GTGTCATAATAAACATGGGAGCATAGAAGC-3′. *Hdac3*^*fl/fl*^ mice were genotyped using the following PCR primer pair: 1597B, 5′-GGACACAGTCATGACCCGGTC-3′; 1133T, 5′-CTCTGGCTTCTGCTATGTCAATG-3′. The floxed allele of *Hdac3*^*fl/fl*^ mice produced a 504-bp PCR product, whereas the wild type allele resulted in a 464-bp PCR product.

### Cell culture

MH-S cells (ATCC, CRL-2019) were cultured with RPMI 1640 with 4.5 g/L D-glucose (Gibco/Thermo Fisher Scientific), 10% FBS, 0.05 mM 2-Mercaptoethanol (Gibco/Thermo Fisher Scientific), and 1% penicillin-streptomycin-amphotericin B at 37 °C in 5% CO_2_. The cell line was passaged at approximately 80–90% confluence.

### Isolation of embryonic and adult cells

Pregnant females were sacrificed by CO_2_ exposure. Embryos ranging from embryonic days E10.5-E18.5 were removed from the uterus and washed in 4 °C phosphate-buffered saline ([PBS], Invitrogen, Carlsbad, CA). Embryos were exsanguinated through decapitation. Lungs, liver, brain, kidneys, spleen, and pancreas were collected, minced into tiny pieces, and incubated for 30 min in PBS containing 1 mg/ml collagenase D (Roche, Basel, Switzerland), 0.01% DNase I (Worthington Biochemical Corp., Lakewood, NJ), and 3% FBS (Hyclone, San Angelo, TX) at 37 °C. Erythrocytes from all the samples were lysed for 3 min with 0.83% NH_4_Cl buffer. The cells from the adult tissues were isolated by the same method with some modifications. The adult lungs were harvested, minced, and incubated in PBS containing 1 mg/ml collagenase D (Roche), 0.01% DNase I (Worthington), and 3% FBS (Hyclone) at 37 °C for 45 min. The erythrocytes were lysed as described above. All the cell suspensions were passed through a 70 μm cell strainer (BD Biosciences, San Jose, CA).

### Flow cytometry and cell sorting

Single-cell suspensions were centrifuged at 450 × *g* for 7 min, resuspended in ice-cold staining buffer (1× PBS with 2% FBS), and placed in 96-well round-bottom plates. After incubation with purified anti-FcγRII/III antibody (clone 2.4G2) at 4 °C for 15 min, cells were stained with a mixture of fluorescent surface antibodies at 4 °C for 30 min. The stained samples were either directly analyzed by flow cytometry or fixed with 2% formalin at 4 °C for 20 min prior to storage at 4 °C overnight. The full list of antibodies used can be found in Supplementary Table 1. Flow cytometry was performed with FACSAria™ II or FACSCelesta™ flow cytometer (BD Biosciences). Data were acquired using BD FACSDiva software version 8.0.2 (BD Biosciences) and analyzed using FlowJo 10.5.3 (BD Biosciences).

The CD45^+^CD11c^hi^Siglec-F^hi^ lung AMs of adult *Hdac3*^fl/fl^ and *Ubc*^*CreER*^*Hdac3*^fl/fl^ mice were sorted by FACS Aria II flow cytometer. The CD45^+^F4/80^hi^CD11b^int^ fetal lung macrophages at E14.5, CD45^+^F4/80^int^CD11b^int^preAMs at E16.5 and E18.5, CD45^+^CD11c^hi^Siglec-F^hi^ adult lung AMs, as well as CD45^+^F4/80^int^CD11b^hi^ FL monocytes at E16.5 from *Hdac3*^fl/fl^ and *Csf1r*^iCre^*Hdac3*^fl/fl^ mice were also sorted by FACSAria™ II flow cytometer. The purity of the isolated populations was >95%.

### Mitochondria function and cell apoptosis assays

The MitoTracker Orange (Invitrogen/Thermo Fisher Scientific) stock solution was prepared according to the manufacturer’s instructions. Briefly, 50 μg of lyophilized MitoTracker Orange dye was dissolved in DMSO to a final concentration of 1 mM and stored at −20 °C in the dark. Cells were incubated with 50 nM MitoTracker Orange in the prewarmed RPMI 1640 medium at 37 °C with 5% CO_2_ for 15 min_,_ followed by two washes with PBS. The stained cells were analyzed by flow cytometry (Ex 554 nm, Em 576 nm).

Cell apoptosis was measured using the Annexin V Apoptosis Detection Kit (eFuor^TM^450 or APC, eBioscience/Thermo Fisher Scientific). In brief, freshly-isolated cells were washed once with 1× binding buffer and resuspended in 100 μL of 1× Binding Buffer at 5 × 10^6^/ml. After adding 3 μL of fluorochrome-conjugated Annexin V, the cells were incubated at RT for 13 min. The stained cells were washed once with 1× binding buffer and analyzed by flow cytometry.

### Tamoxifen treatment

Tamoxifen (Sigma Aldrich, St. Louis, MO) was dissolved in corn oil (Sigma Aldrich) containing 10% (vol/vol) ethanol (Thermo Fisher Scientific) and was intraperitoneally administered at 50 mg/kg of mouse weight for five consecutive days.

### Bone marrow chimeras

B6.SJL^Hetero^ (CD45.1^+^CD45.2^+^) mice were lethally irradiated once with 950 rads. Donor BM cells were harvested from age-matched and sex-matched CD45.2^+^ *Hdac3*^*fl/fl*^ and *Csf1r*^*iCre*^*Hdac3*^*fl/fl*^ mice, as well as B6.SJL (CD45.1^+^) mice. The BM cells from *Hdac3*^*fl/fl*^ or *Csf1r*^*iCre*^*Hdac3*^*fl/fl*^ mice were mixed with the cells from B6.SJL mice (at a 4:1 ratio) and then co-transferred to the irradiated B6.SJL^Hetero^ recipient mice (10 × 10^6^ mixed cells/mouse). The recipient mice were sacrificed 10 wks after reconstitution.

### RNA extraction and quantitative real-time PCR

Total RNA was extracted from TRMs with miRCURY RNA Isolation Kit—Cell and Plant (Exiqon). The RNA was reverse-transcribed to cDNA with High Capacity cDNA Reverse Transcription Kits (Applied Biosystems, Foster City, CA). Quantitative real-time PCR (qRT-PCR) reactions were prepared using FastStart Universal SYBR Green Master (ROX, Roche) and carried out using QuantStudio 7 Flex Real-Time PCR System (Applied Biosystems). Data were collected using QuantStudio 7 Flex Real-Time PCR System software version 1.2 (Applied Biosystems) and analyzed using Microsoft Excel 2016 (Microsoft, Redmond, Washington). The full list of primers used can be found in Supplementary Table 2. Samples were normalized to GAPDH expression.

### Immunofluorescence

A piece of lung or liver freshly isolated from adult mice or embryos at E18.5 was immediately frozen in OCT compound for cryostat sectioning (5 μm per section). The frozen tissue sections were fixed with acetone for 15 min at −20 °C. After rinsing with PBS for 5 min, samples were blocked with anti-FcγRII/III antibody at RT for 15 min followed by staining at 4 °C overnight with PE-labeled anti-Siglec-F (adult lung) or PE-labeled anti-F4/80 (fetal lung) antibody. The samples were then stained with 1 μg/ml DAPI at RT for 1 min and mounted under a coverslip with one drop of IMMU-MOUNT (Thermo Fisher Scientific). Specimens were visualized on an Olympus FSX100 fluorescence microscope (Olympus Scientific Solutions Americas Corp, Waltham, MA) at ×10 magnification. At least 5 independent images were collected from each specimen. Images were collected using FSX-BSW (version 03.02.12) software (Olympus). The cell density of AMs (Siglec-F^+^) or preAMs/TRMs (F4/80^+^) was measured based on cell numbers counted in the fields using cellSens Dimensions Imaging Software version 1.15 (Olympus).

### Bulk RNA sequencing

To achieve optimal library quality, cDNA synthesis and preamplification for total RNA of the sorted lung preAMs from WT or *Csf1r*^*iCre*^*Hdac3*^*fl/fl*^ cKO embryos at E18.5 were performed using the SMART-Seq v4 Ultra Low Input RNA Kit (Clontech, Mountain View, CA) following the manufacturer’s instructions. Briefly, cDNA was pre-amplified by a thermal cycler (Applied Biosystem) using 12–14 cycles and sheered into 150–400 bp fragments using a Bioruptor Pico sonicator (Diagenode, Denville, NJ). The DNA ends were repaired using End-It™ DNA End-Repair Kit (Epicentre, Charlotte, SC). The end-repaired DNA fragments were ligated to adapters by T4 ligase (New England Bioloabs, Ipswich, MA) after a single A was added at 3′ by Klenow Fragment (3′ → 5′ Exo-) (New England Bioloabs). MinElute Reaction Cleanup Kit (Qiagen, Gaithersburg, MD) was used for DNA cleanup after each step. Illumina P5 and P7 primers with indicated adapters (Supplementary Table 3) were used for final library amplification. The 150–400 bp final product was purified on a 2% E-gel (Thermo Fisher Scientific). Sequencing was performed by the DNA sequencing core facility at University of Michigan using a 50 bp single end read setup on the Illumina Hiseq 4000 platform.

### Single-cell RNA sequencing

Single-cell sequencing libraries were generated by an established protocol using the 10× Genomics Chromium Single Cell 3′ Reagent Kit (v2 Chemistry) and Chromium Single Cell Controller^59^. Briefly, 5000 FACS-sorted cells were loaded into each reaction for gel bead-in-emulsion (GEM) generation and cell barcoding. Reverse transcription of the GEM (GEM-RT) was performed using a Veriti™ 96-Well Fast Thermal Cycler, (Applied Biosystems) at 53 °C for 45 min, 85 °C for 5 min, and a 4 °C hold. cDNA amplification was performed after GEM-RT cleanup with Dynabeads MyOne Silane (Thermo Fisher Scientific) using the same thermocycler (98 °C for 3 min, 98 °C for 15 s, 67 °C for 20 s, 72 °C for 1 min, followed by a 12 cycle repeat at 72 °C for 1 min and a 4 °C hold). Amplified cDNA was cleaned up with SPRIselect Reagent Kit (Beckman Coulter, Brea, CA) that was followed by a library construction procedure, including fragmentation, end repair, adapter ligation, and library amplification. An Agilent 2100 Bioanalyzer (Agilent, Santa Clara, CA) was used for library quality control. Libraries were sequenced on an Illumina HiSeq4000 using a paired-end flow cell: Read 1, 26 cycles; i7 index, 8 cycles; Read 2, 98 cycles.

### ChIP sequencing

Cell pellets from freshly-isolated AMs (1.38 × 10^6^ and 0.77 × 10^6^ cells for WT and cKO, respectively) of adult *Hdac3*^*fl/fl*^ WT (*n* = 13) or *Csf1r*^*iCre*^*Hdac3*^*fl/fl*^ cKO mice (*n* = 26) were directly frozen at −80 °C. The samples were then submitted to EpiGentek (Farmingdale, NY) for ChIP, library preparation, and sequencing. Briefly, the cells were fixed with 1% formaldehyde and chromatin was isolated using a ChromaFlash^TM^ Chromatin Extraction Kit. Chromatin was sheared using the Episonic2000 Sonicatin System. ChIP was performed using an antibody against HDAC3 (Abcam, #ab7030, polyclonal, 2 µg/500 µl assay buffer) on the chromatin, and input DNA (without immunoprecipitation) was used as background. After adapter linking, the library was size selected (100–300 bp) and PCR amplified. Ten nanomolar of each sample library was provided for next generation sequencing on a HiSeq 4000.

### ChIP-qPCR

The ChIP assay was performed on 80–90% confluent cultures using an EZ-ChIP kit (Millipore, Burlington, MA, #17-371) and 1 μg of anti-RNA Polymerase II antibody (Millipore, #05-623B, clone CTD4H8, 1 μg/ml), 2 μg of anti-HDAC3 antibody (Abcam, #ab32369, clone Y415, 2 μg/ml), 1 μg of anti-PPAR-γ antibody (Cell Signaling, Danvers, MA, #2443 S, clone 81B8, 1 μg/ml), and 1 μg of rabbit IgG (Abcam, #ab171870, polyclonal, 1 μg/ml) per reaction. DNA-relative enrichment was determined by normalization to an input genomic DNA. All ChIP experiments were obtained from independent chromatin preparations, and all quantitative real-time PCR reactions were performed in duplicates for each sample on each amplicon. Primers for the ChIP-qPCR are listed in Supplementary Table 2.

### Bulk RNA-Seq and ChIP-Seq data processing

The RNA-Seq reads were aligned to the mouse GRCm38/mm10 reference genome and expression levels for each sample were quantified and normalized using Biomedical Genomics Workbench 5.0 (Qiagen). Gene ontology (GO) biological process enrichment analysis was performed using DAVID Bioinformatics Resources 6.8^60^. Gene Set Enrichment Analysis (GSEA) was performed using GSEA software 4.0.3^61,62^. Further pathway analysis was performed using the Ingenuity Pathway Analysis ([IPA], Qiagen Bioinformatics, version 42012434). Raw ChIP-seq reads were quality checked using the software FastQC version v0.10.1^63^ and processed using BBDuk (v36.19) and seqtk (v1.2-r94, https://github.com/lh3/seqtk) to trim the adapters and low-quality bases, respectively. The trimmed reads were then aligned to the mouse mm10 genome sequence using Bowtie2 (v2.2.5)^64^ and only uniquely-matching reads were retained. Mapping results of each ChIP and the input sample were subjected to ChIP-enriched peak calling using the MACS2^65^ (v2.1.1.20160309). Peak annotation was conducted using ChIPseeker (v1.24.0)^66^.

### scRNA-seq data processing

Paired-end read counts for all known murine genes underwent barcode and unique molecular identifier (UMI) deconvolution. They were subsequently aligned to the mm10 reference genome using the 10× Cell RangerTM [v2.1] pipeline^67,68^. A total of 2000–3000 cells were captured, with 53,000–73,000 mean reads per cell, and 1200–1300 median genes per cell. Datasets were subsequently analyzed using R-3.3.2 package Seurat’s Canonical Correlation Analysis (CCA) workflow to characterize the differences between pooled samples^69^.

The following quality control metrics were employed. To identify potential doublets, cells expressing uncharacteristically high numbers of genes (>2500) were excluded (double the median number of genes). Low-quality cells were excluded based on a low number of genes detected (<300) or having high mitochondria genetic content (>5.0%). In addition, uninteresting sources of variation within the data were removed. Genes removed include sex-specific genes (Xist and all Y chromosome genes), ribosomal structural proteins as identified by gene ontology term GO:0003735 and the RPG: Ribosomal Protein Gene database^70^, non-coding rRNAs, Hbb, and genes expressed in <3 cells.

The global-scaling normalization method, LogNormalize, was employed to normalize gene expression measurements of each cell by the total expression, multiplying this by a factor of 10,000, followed by log-transformation. Highly variable genes in each data analysis were identified, and the intersecting top 500 genes in each dataset were used for clustering and downstream analyses. Datasets underwent scaling based on nUMI and percent mitochondria gene content. The number of CCs (1:15) used to cluster cells was determined by manual inspection of scree plots. Further pathway analysis was performed using IPA.

### Statistics and reproducibility

No statistical method was utilized to predetermine sample size. Student’s unpaired two-tailed *t* tests were performed using GraphPad Prism 8.4.3 software (GraphPad, La Jolla, CA) unless otherwise specified. Statistical significance is displayed as: *N.S*., not significant; *P < 0.05; **P < 0.01; ***P < 0.001.

Bulk RNA-seq (three biologically independent samples/group), scRNA-seq (single sample/group), and ChIP-seq (single sample/group) were performed one time. Immunofluorescence, bone marrow chimera, ChIP-qPCR and qRT-PCR experiments were performed twice independently with similar results. All other experiments were performed at least three times independently with similar results.

### Reporting summary

Further information on research design is available in the Nature Research Reporting Summary linked to this article.

## Supplementary information

Supplementary Information

Description of Additional Supplementary Information

Supplementary Data 1

Supplementary Data 2

Supplementary Data 3

Reporting Summary

## Data Availability

All ChIP-seq, bulk RNA-seq, and scRNA-seq data reported here have been deposited in the Gene Expression Omnibus (GEO) under accession number SuperSeries GSE122533. The data of PPAR-γ microarray (GSE60249^7^) had been previously disclosed in GEO by other researches. DAVID Bioinformatics Resources 6.8^60^ and RPG: Ribosomal Protein Gene database^70^ are both publicly accessible. The data supporting this study are available in the Article, Supplementary Information, Source Data, or available from the authors upon reasonable requests. The reporting summary and editorial checklist for this article are available as a Supplementary file. Source data are provided with this paper.

## Source data

Source Data
