# Supplementary material for: Histone deacetylase 3 controls lung alveolar macrophage development and homeostasis
Source: Nat Commun. 2020 Jul 30;11:3822. doi: 10.1038/s41467-020-17630-6 (PMC7393351; doi:10.1038/s41467-020-17630-6)
Supplement: Supplementary file 1 — Supplementary Information [file 41467_2020_17630_MOESM1_ESM.pdf]

Supplementary Information

Histone Deacetylase 3 controls lung alveolar macrophage development and homeostasis

Yao et al.

Supplementary Figure 1

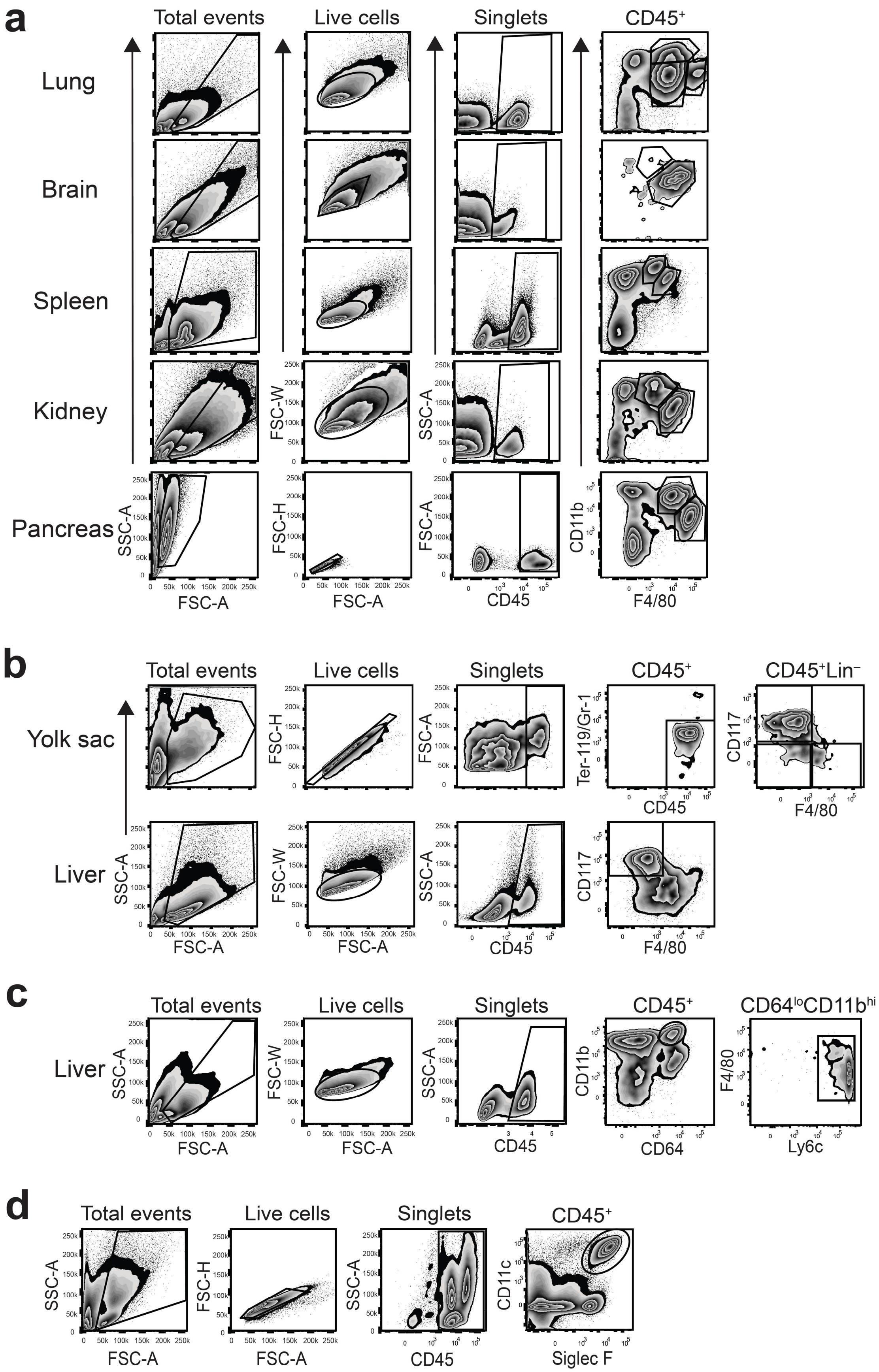

Supplementary Figure 1

Gating strategy for adult AMs, fetal preAMs, TRMs, and their YS or FL progenitors.

(a) Single-cell suspensions of lung and other indicated organs were prepared from *Hdac3*$^{fl/fl}$ embryos at E18.5. After excluding dead cells (FSC-A vs. SSC-A) and doublets (FSC-A vs. FSC-H), all cells were gated based on CD45 expression. CD45^{+} cells were further gated based on F4/80 and CD11b expression for preAMs (F4/80^{int}CD11b^{int}), TRMs (F4/80^{hi}CD11b^{int}), and monocytes (F4/80^{int}CD11b^{hi}). Gating strategy for sorting and analysis of lung TRMs and preAMs (Fig. 1a-d, Fig. 5a-k, Fig. 6a-c,f-h, Fig. 7a-f, h-j, Supplementary Fig. 7a,b, 8a,b) and for analysis of TRMs and monocytes in other organs (Supplementary Fig. 3a,b). **(b)** CD117 and F4/80 expression was analyzed for EMPs (CD117^{hi}F4/80$^{–}$), pMacs (CD117$^{–}$F4/80$^{–}$), and macrophages (CD117$^{–}$F4/80^{+}) within CD45^{+}Lin$^{–}$ live singlets in YS at E9.5. EMPs were also analyzed within CD45^{+} live cells in FL at E12.5. Gating strategy for data presented on Fig. 1g. (**c**) FL monocytes (CD64^{lo}CD11b^{hi}Ly6c^{hi}) were analyzed within CD45^{+} live cells at E18.5. >97% of CD64^{lo}CD11b^{hi} cells were Ly6c^{hi}. Gating strategy for data presented on Fig. 1i and Supplementary Fig. 2a,b. **(d)** Gating strategy used to sort and analyze lung AMs (CD11c^{hi}Siglec F^{hi}) within CD45^{+} live cells from *Hdac3*$^{fl/fl}$ adults (for data presented on Fig. 2a,c-f, Fig. 3b, Fig. 4a-f, Fig. 6d, Supplementary Fig. 4a,b, Fig.5, Fig. 6a-c). Representative data shown are from at least 3 independent experiments.

Supplementary Figure 2

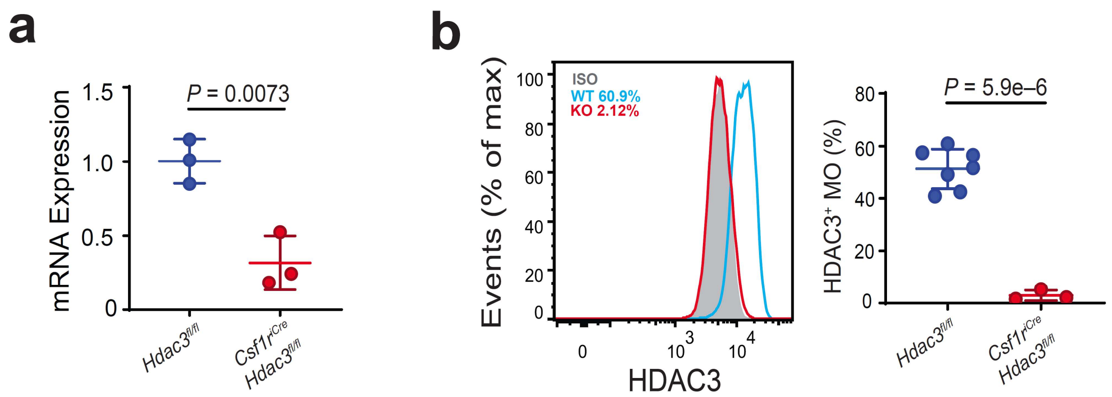

Supplementary Figure 2

Deletion of HDAC3 in FL monocytes from HDAC3-deficient mice.

(**a**) qRT-PCR analysis of HDAC3 mRNA expression in FL monocytes (MO) isolated from the *Hdac3*$^{fl/fl}$ and *Csf1r*^{Cre}*Hdac3*$^{fl/fl}$ embryos at E16.5. Fold change represents the relative expression levels of HDAC3 compared to WT *Hdac3*$^{fl/fl}$ mice. Bars represent mean ± SD from three biologically independent samples per group. (**b**) Flow cytometry of HDAC3 expression on FL MO from *Hdac3*$^{fl/fl}$ and *Csf1r*^{icre}*Hdac3*$^{fl/fl}$ newborns (P0-P1). Rabbit IgG served as an isotype control (Iso). Frequencies of HDAC3^{+} FL MO from *Hdac3*$^{fl/fl}$ (n = 7) and *Csf1r*^{icre}*Hdac3*$^{fl/fl}$ (n = 3) biologically independent samples shown on the right panel. One dot presents one mouse and bars represent mean ± SD. All *P* values were obtained using the Student's two-tailed unpaired *t* test. Source data are provided as a Source Data file.

Supplementary Figure 3

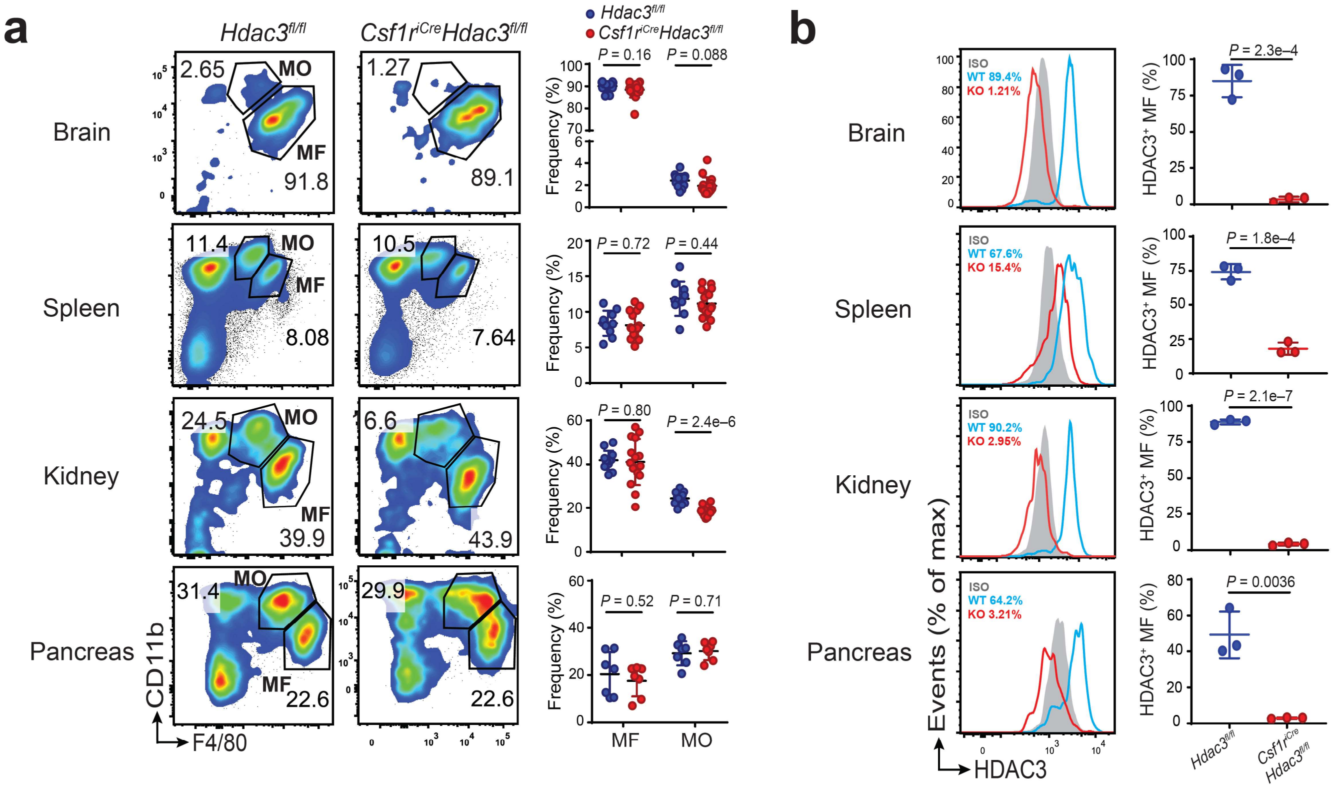

Supplementary Figure 3

TRMs and MOs in brain, spleen, kidney, and pancreas from HDAC3-deficient embryos.

(**a**) Flow cytometry analysis of macrophages (MF, F4/80^{hi}CD11b^{int}) and monocytes (MO, F4/80^{int}CD11b^{hi}) of indicated organs from *Hdac3$^{fl/fl}$* and *Csf1r^{Cre}Hdac3$^{fl/fl}$* embryos at E18.5. The frequencies of MF and MO (mean ± SD) are shown on the right. Brain: *Hdac3$^{fl/fl}$* (n = 12) and *Csf1r^{Cre}Hdac3$^{fl/fl}$* (n = 17) biologically independent samples; spleen: *Hdac3$^{fl/fl}$* (n = 10) and *Csf1r^{Cre}Hdac3$^{fl/fl}$* (n = 15) biologically independent samples; kidney: *Hdac3$^{fl/fl}$* (n = 12) and *Csf1r^{Cre}Hdac3$^{fl/fl}$* (n = 15) biologically independent samples; pancreas: *Hdac3$^{fl/fl}$*;*Csf1r^{Cre}Hdac3$^{fl/fl}$* (n = 7) biologically independent samples. (**b**) Flow cytometry of HDAC3 expression on MFs of indicated organs from *Hdac3$^{fl/fl}$* and *Csf1r^{icre}Hdac3$^{fl/fl}$* newborns (P0-P1). Frequencies of HDAC3^{+} MF (mean ± SD) shown on the right panel. One dot presents a pooled sample from 1-3 mice. n = 3 *Hdac3$^{fl/fl}$*;*Csf1r^{Cre}Hdac3$^{fl/fl}$* biologically independent samples for each organ. All *P* values were obtained using the Student's two-tailed unpaired *t* test. Source data are provided as a Source Data file.

Supplementary Figure 4

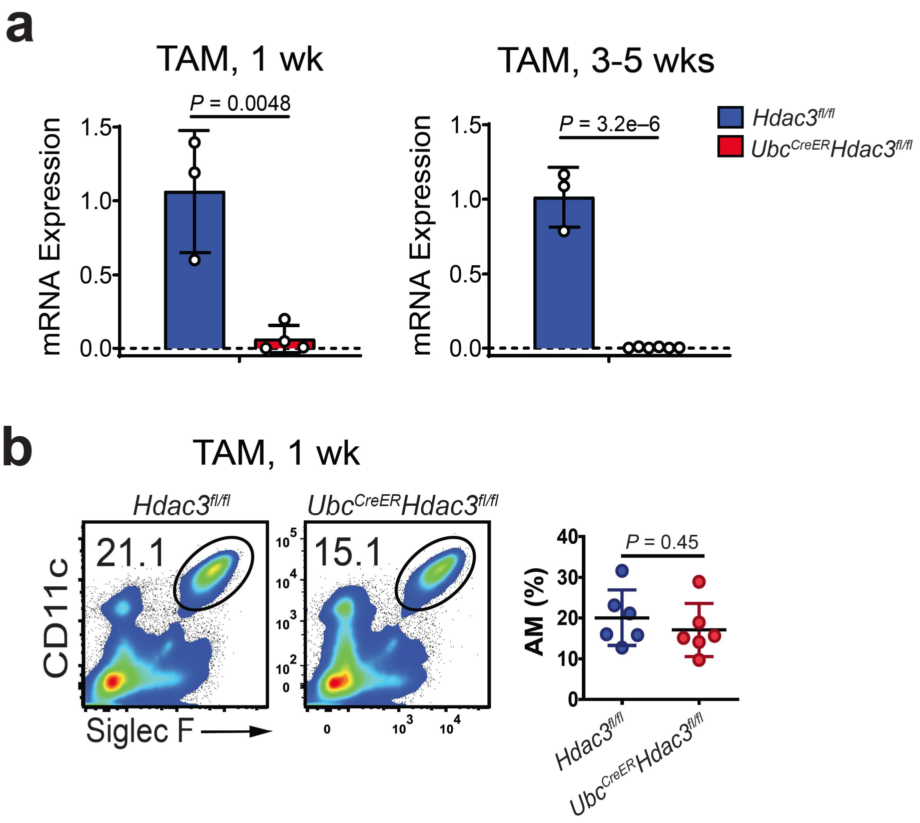

Supplementary Figure 4

AM maintenance in *$Ubc^{CreER}Hdac3^{fl/fl}$* adult mice following inducible deletion of HDAC3.

$Hdac3^{fl/fl}$ and *$Ubc^{CreER}Hdac3^{fl/fl}$* adult mice were administered intraperitoneally with tamoxifen (TAM, 50 mg/kg body weight per day) for 5 consecutive days. Mice were sacrificed for collection of lung tissues at 1 wk or 3-5 wks after the last dose of TAM treatment. (**a**) qRT-PCR analysis of HDAC3 mRNA expression in AMs freshly isolated from the TAM-treated mice (mean ± SD, 1 wk: *$Hdac3^{fl/fl}$* (n = 3) and *$Ubc^{CreER}Hdac3^{fl/fl}$* (n = 4) biologically independent samples; 3-5 wks: *$Hdac3^{fl/fl}$* (n = 3) and *$Ubc^{CreER}Hdac3^{fl/fl}$* (n = 6) biologically independent samples. (**b**) Flow cytometry analysis of AMs isolated from the mice at 1 wk after TAM treatment. The frequencies of AMs are shown as mean ± SD from *$Hdac3^{fl/fl}$*;*$Ubc^{CreER}Hdac3^{fl/fl}$* (n = 6) biologically independent samples. All *P* values were obtained using the Student's two-tailed unpaired *t* test. Source data are provided as a Source Data file.

Supplementary Figure 5

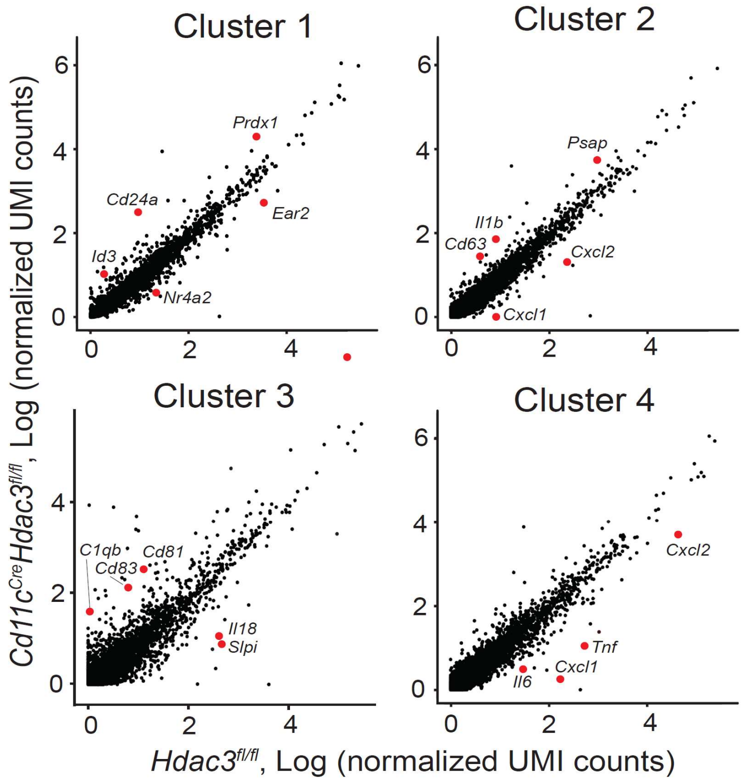

Supplementary Figure 5

HDAC3-regulated transcriptome changes in AM subsets.

CD11c^{hi}Siglec-F^{hi} lung AMs were sorted from *Hdac3*$^{fl/fl}$ and *Cd11c*^{cre}*Hdac3*$^{fl/fl}$ adult mice for scRNA-seq analysis. Scatter plot depicting genes differentially expressed between two groups in each AM subset.

Supplementary Figure 6

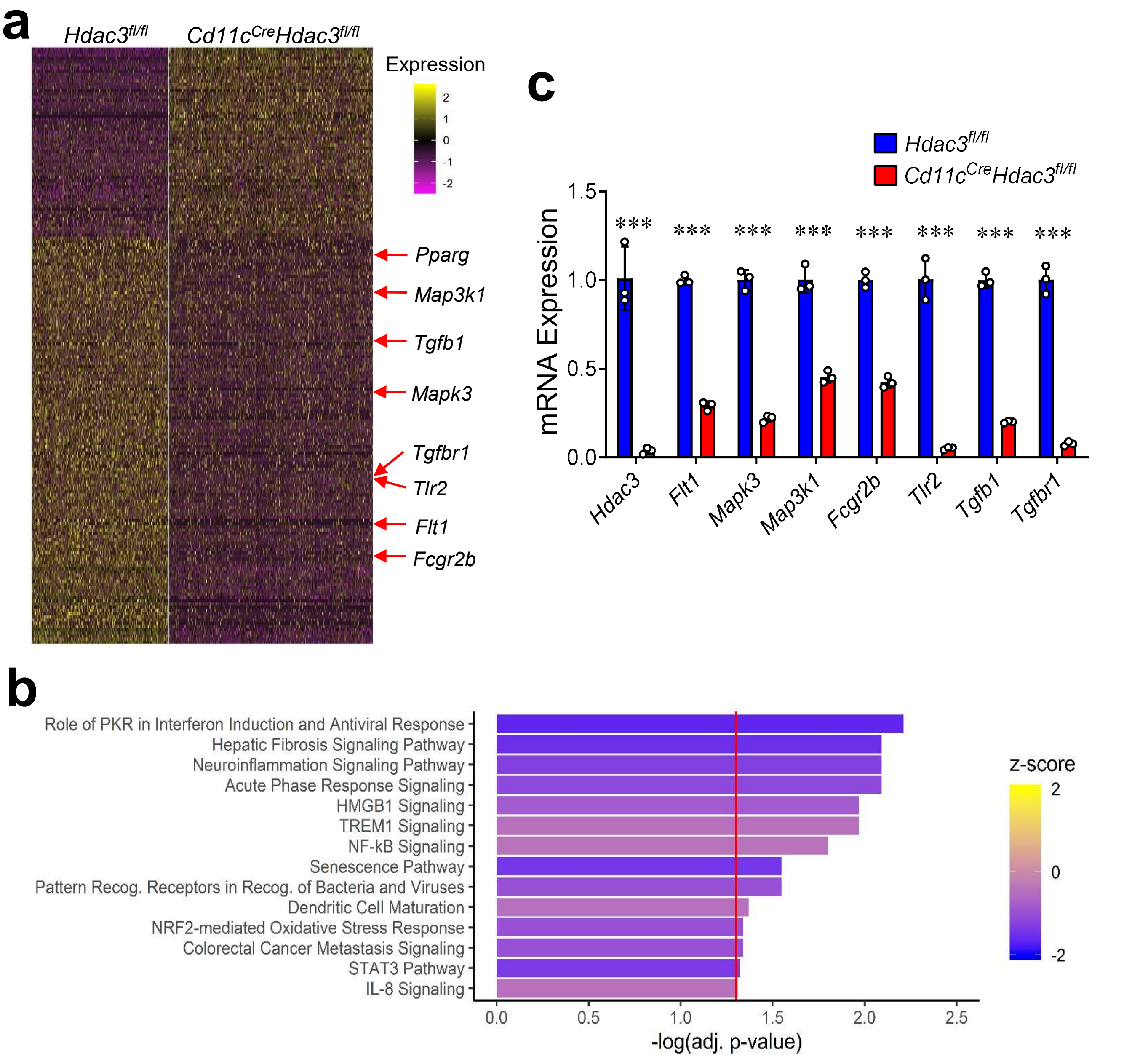

Supplementary Figure 6

HDAC3-regulated transcriptome changes in AM populations.

CD11c^{hi}Siglec-F^{hi} lung AMs were sorted from *Hdac3*$^{fl/fl}$ and *Cd11c*^{cre}*Hdac3*$^{fl/fl}$ adult mice for scRNA-seq analysis. (**a**) Heat map showing genes differentially expressed between two groups. (**b**) IPA analysis of canonical pathways inhibited in the AMs from *Cd11c*^{cre}*Hdac3*$^{fl/fl}$ mice mice. Adjusted P value < 0.05 by the right-tailed Fisher's exact test with the Benjamini-Hochberg correction for multiple testing; Z-score < 0. (**c**) qRT-PCR analysis of dysregulated genes related to TREM1, NF-kB and STAT3 pathways from *Hdac3*$^{fl/fl}$;*Cd11c*^{Cre}*Hdac3*$^{fl/fl}$ (n = 3) biologically independent samples (mean ± SD). ****P* = 7.3e–4 (*Hdac3*), 3.9e–6 (*Flt1*), 2.2e–5 (*Mapk3*), 3.4e–4 (*Map3k1*), 5.2e–5 (*Fcgr2b*), 1.5e–4 (*Tlr2*), 4.8e–6 (*Tgfb1*), 3.8e–5 (*Tgfbr1*) by the Student's two-tailed unpaired *t* test. Source data are provided as a Source Data file.

Supplementary Figure 7

a

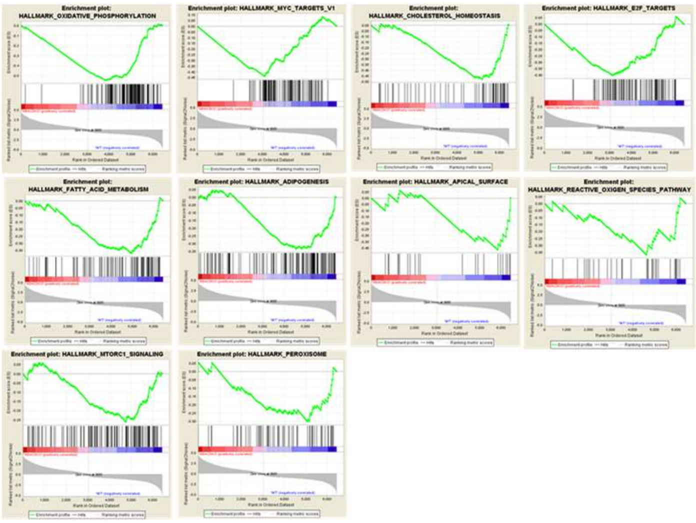

b

NAME	SIZE	ES	NES	NOM p-val	FDR q-val	RANK AT MAX
HALLMARK_OXIDATIVE_PHOSPHORYLATION	158	-0.55	-3.31	0	0	2561
HALLMARK_MYC_TARGETS_V1	115	-0.44	-2.49	0	5.2E-04	3335
HALLMARK_CHOLESTEROL_HOMEOSTASIS	57	-0.48	-2.35	0	9.1E-04	1235
HALLMARK_E2F_TARGETS	100	-0.40	-2.24	0	9.5E-04	3304
HALLMARK_FATTY_ACID_METABOLISM	97	-0.37	-2.05	0	9.4E-04	1444
HALLMARK_ADIPOGENESIS	136	-0.34	-1.98	0	9.6E-04	1853
HALLMARK_REACTIVE_OXIGEN_SPECIES_PATHWAY	31	-0.37	-1.59	0.028	4.0E-02	1785
HALLMARK_APICAL_SURFACE	22	-0.41	-1.57	0.045	3.7E-02	587
HALLMARK_MTORC1_SIGNALING	132	-0.26	-1.56	0.010	3.6E-02	1682
HALLMARK_PEROXISOME	59	-0.30	-1.51	0.014	4.5E-02	1352

Supplementary Figure 7

GSEA analysis of downregulated genes in the fetal preAMs from *Csf1r*^{iCre}*Hdac3*$^{fl/fl}$ mice.

(**a**) Enrichment plots for gene sets with negative NES and FDR below 0.05. (**b**) The table presents the enriched gene sets with the number of genes belonging to each gene set (SIZE), their enrichment scores (ES), normalized enrichment scores (NES), nominal *P*-values (NOM p-val), FDR q-values (FDR q-val), and the position in the ranked list at which the maximum enrichment score occurred (RANK AT MAX). The statistical methods were used by GSEA algorithm: ES computed based on two-tailed weighted Kolmogorov-Smirnov test; the significant of ES (nominal *P*-values) estimated using an empirical phenotype-based permutation test procedure; FDR q-values calculated with Benjamini-Yekutieli correction for multiple testing.

Supplementary Figure 8

a

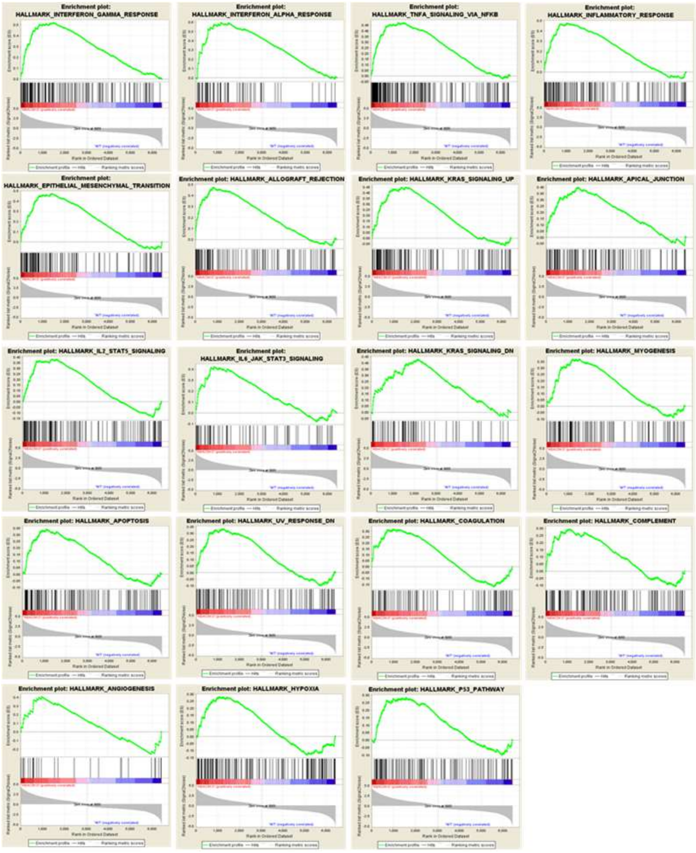

b

NAME	SIZE	ES	NES	NOM p-val	FDR q-val	RANK AT MAX
HALLMARK_INTERFERON_GAMMA_RESPONSE	144	0.52	2.83	0	0	1491
HALLMARK_INTERFERON_ALPHA_RESPONSE	67	0.60	2.76	0	0	1143
HALLMARK_TNFA_SIGNALING_VIA_NFKB	158	0.47	2.56	0	0	1246
HALLMARK_INFLAMMATORY_RESPONSE	141	0.48	2.50	0	0	1029
HALLMARK_EPITHELIAL_MESENCHYMAL_TRANSITION	125	0.47	2.44	0	0	1417
HALLMARK_ALLOGRAFT_REJECTION	111	0.48	2.42	0	0	813
HALLMARK_KRAS_SIGNALING_UP	122	0.45	2.33	0	0	1351
HALLMARK_APICAL_JUNCTION	115	0.40	2.06	0	6.2E-04	1462
HALLMARK_IL2_STAT5_SIGNALING	140	0.38	2.03	0	5.5E-04	1370
HALLMARK_KRAS_SIGNALING_DN	66	0.43	2.03	0	4.9E-04	2166
HALLMARK_IL6_JAK_STAT3_SIGNALING	66	0.42	1.97	0	6.9E-04	848
HALLMARK_MYOGENESIS	106	0.37	1.87	0	1.7E-03	1522
HALLMARK_APOPTOSIS	125	0.34	1.79	0	3.5E-03	1102
HALLMARK_UV_RESPONSE_DN	101	0.33	1.69	0.001	1.0E-02	1271
HALLMARK_COAGULATION	87	0.32	1.58	0.009	2.5E-02	949
HALLMARK_COMPLEMENT	127	0.30	1.56	0.015	2.8E-02	1102
HALLMARK_ANGIOGENESIS	28	0.41	1.53	0.036	3.2E-02	957
HALLMARK_HYPOXIA	142	0.28	1.49	0.014	4.0E-02	1086
HALLMARK_P53_PATHWAY	123	0.28	1.48	0.018	4.1E-02	1562

Supplementary Figure 8

GSEA analysis of upregulated genes in the fetal preAMs from *Csf1r^{iCre}Hdac3$^{fl/fl}$* mice.

(**a**) Enrichment plots for gene sets with positive NES and FDR below 0.05. (**b**) The table presents the enriched gene sets with the number of genes belonging to each gene set (SIZE), their enrichment scores (ES), normalized enrichment scores (NES), nominal *P*-values (NOM p-val), FDR q-values (FDR q-val), and the position in the ranked list at which the maximum enrichment score occurred (RANK AT MAX). The statistical methods were used by GSEA algorithm: ES computed based on two-tailed weighted Kolmogorov-Smirnov test; the significant of ES (nominal *P*-values) estimated using an empirical phenotype-based permutation test procedure; FDR q-values calculated with Benjamini-Yekutieli correction for multiple testing.

Supplementary Figure 9

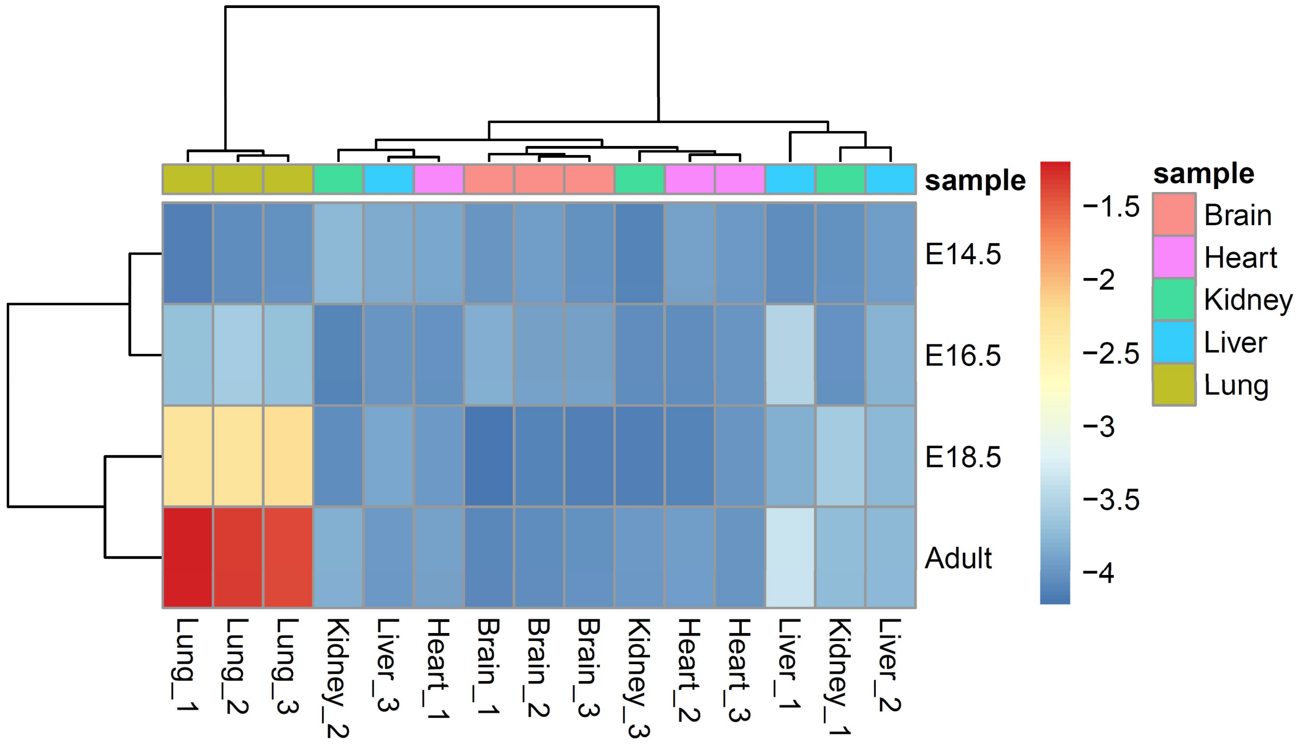

Supplementary Figure 9

PPAR-γ mRNA expression profile during TRM development.

Heat map representation of mRNA expression of PPAR-γ in TRMs of lung, kidney, liver, brain, and heart from C57BL/6 mice at E14.5, E16.5, E18.5 and adulthood determined by qRT-PCR. n = 3 biologically independent samples for each organ and time point. Data were normalized to GAPDH expression (delta Ct) for individual sample. Color scale for expression values of –Log_2(delta Ct). Source data are provided as a Source Data file.

Supplementary Table 1

Antibody	Clone	Conjugate	Company	Catalog#	Concentration
CD11b	M1/70	PerCP/Cy5.5	eBioscience	45-0112-82	0.05 µg/100 µL
CD11b	M1/70	APC-CY7	Biolegend	101226	0.1 µg/100 µL
CD11b	M1/70	FTIC	eBioscience	11-0112-85	0.25 µg/100 µL
CD11c	N418	PE	eBioscience	12-0114-83	0.1 µg/100 µL
CD11c	N418	APC	eBioscience	17-0114-82	0.1 µg/100 µL
CD11c	N418	PerCP/Cy5.5	Biolegend	117328	0.1 µg/100 µL
CD11c	N418	ef450	eBioscience	48-0114-82	0.05 µg/100 µL
CD11c	N418	PE-Cy7	Biolegend	117318	0.1 µg/100 µL
CD117 (c-Kit)	2B8	PerCP/Cy5.5	BioLegend	105824	0.2 µg/100 µL
CD117 (c-Kit)	2B8	PE	eBioscience	12-1171-82	0.05 µg/100 µL
CD207 (langerin)	4C7	PE	Biolegend	144204	0.1 µg/100 µL
CD207 (langerin)	4C7	APC	Biolegend	144206	0.2 µg/100 µL
CD45	30-F11	APC	Tonbo Biosciences	20-0451-U100	0.01 µg/100 µL
CD45	30-F11	FITC	Tonbo Biosciences	35-0451-U500	0.5 µg/100 µL
CD45	30-F11	PE	Tonbo Biosciences	50-0451-U100	0.1 µg/100 µL
CD45	30-F11	APC-Cy7	BioLegend	103116	0.05 µg/100 µL
CD45.1	A20	APC	Tonbo Biosciences	20-0453-U100	0.1 µg/100 µL
CD45.1	A20	FITC	Tonbo Biosciences	35-0453-U500	0.1 µg/100 µL
CD45.1	A20	APC-ef780	eBioscience	47-0453-82	0.2 µg/100 µL
CD45.2	104	PE	eBioscience	12-0454-82	0.2 µg/100 µL
CD45.2	104	PerCP/Cy5.5	eBioscience	45-0454-82	0.1 µg/100 µL
CD45.2	104	FITC	Tonbo Biosciences	35-0454-U500	0.25 µg/100 µL
CD64	X54-5/7.1	PE	eBioscience	12-0641-82	0.05 µg/100 µL
F4/80	BM8	eFluor 450	eBioscience	48-4801-82	0.05 µg/100 µL

F4/80	BM8.1	APC	Tonbo Biosciences	20-4801-U100	0.1 μg/100 μL
I-A/E	M5/114.15.2	PE	eBioscience	12-5321-83	0.02 μg/100 μL
I-A/E	M5/114.15.2	APC	eBioscience	17-5321-82	0.1 μg/100 μL
I-A/E	M5/114.15.2	FITC	eBioscience	11-5321-82	0.05 μg/100 μL
I-A/E	M5/114.15.2	eFluor 450	eBioscience	48-5321-82	0.04 μg/100 μL
Ly6c	H41.4	Alexa Fluor 488	eBioscience	53-5932-82	0.1 μg/100 μL
Siglec F	E50-2440	PE	BD Biosciences	552126	0.1 μg/100 μL
Siglec F	E50-2440	eFluor 660	eBioscience	50-1702-82	0.1 μg/100 μL
Annexin V		APC	eBioscience	17-8007-74	2.0 μL/100 μL

Supplementary Table 1: Antibodies used for flow cytometry

Supplementary Table 2

Gene (Accession)	Forward (5'→3')	Reverse (5'→3')
Gapdh	GGTGAAGGTCGGTGTGAACG	TGTAGACCATGTAGTTGAGGTCA
Hdac3	AGCTGGACACCCAATGAAAC	TGTTGCTCCTTGCAGAGATG
Il1b	GAGGACATGAGCACCTTCTTT	GCCTGTAGTGCAGTTGTCTAA
Il6	TCCAGTTGCCTTCTTGGGAC	GTACTCCAGAAGACCAGAGG
Tnf	ACAAAGGTGCCGCTAACCACATGT	ATGCTGCTGTTTCAGTCGAAGGCA
Pparg	TGGGTGAAACTCTGGGAGATTC	GAGAGGTCCACAGAGCTGATTCC
Ppargc1b	GGTGTTCGGTGAGATTGTAGAG	CTGAACACCGGAAGGTGATAAA
Acsl1	GCTTGTGGATGTGGAAGAAATG	TCTTGCTGGGTCTTTCAAGTAG
Atf3	CTCCTGGGTCACTGGTATTTG	CCGATGGCAGAGGTGTTTAT
Atp5g3	GGGAGTTTCAGACCAGTGTAAT	TCCAATACCAGCACCAGAAC
Atp5l	AGACGTACCTTCCACCTTAGA	CAATCGAGGCTTCGAGTAAGT
Bmp2	AGTAGTTTCCAGCACCGAATTA	TTCACTAACCTGGTGTCCAATAG
Ccnd1	CAGAGGCGGATGAGAACAAG	GAGGGTGGGTTGGAAATGAA
Cd38	GTGGCAGGATTAGAAGGTATGG	TGAAGGCTGTTAGTGGAATAGTG
Cidec	ATGGACTACGCCATGAAGTCT	CGGTGCTAACACGACAGGG
Cox5A	GATGCTCGCTGGGTGACATA	AACCGTCTACATGCTCGCAA
Cox5B	AGAAGGGACTGGACCCATACA	CCTTTGTGCAGCCAAAACCA
Cox6a1	GAGGGTTCAGCTCGGATGTG	GGTCTCTCGTGCTCTTCGTG
Cox6b1	AACTACCTGGACTTCCACCG	GGTACCACTCACACACGGAG
Cox6C	GCCTATAAGTTTGGCGTGGC	TTGGAGCGCAATCCAAAGAA
Cox7B	AATTTGCACCAAGGCAGCAG	CTTCGAACTTGGAGACGGCTT
Fabp4	GTGAAGAGCATCATAACCCTAGAT	CACGCCTTTCATAACACATTCC
Fcgr2b	CTGAGGCTGAGAATACGATCAC	CCAATGCCAAGGGAGACTAAA
Flt1	CCGAATGGCTTTCACGTTTC	GCAGAATCCAGGTAATGTCTCT
Map3k1	AATGTTCCCAGCACCATCA	GAGGAGATGCGGAAGGTATTT
Mapk3	CAGAGTGGCCATCAAGAAGAT	GGATGTCTCGGATGCCTATAAC

Mgst3	GTACAGCACAGATCCTGAGAAC	GTAAACACCTCCCACCGTTAG
Mmp2	GTTCAACGGTCGGGAATACA	GCCATACTTGCCATCCTTCT
Ndufa4	AGCCATGGAACAAACTGGGT	ATTGTGCGGATGGCTTCTGA
Ndufa8	GAGTTTATGCTGTGCCGCTG	TACTCTGTGAAAGGCTCCGC
Ndufb2	TTTGGCATGACTCGGATGCT	TCCTCGTCATCAGGAGGGAT
Nr1h3	TTGCGTAGCATTAAGGGAGAG	GGCTCTCCTGGCTAGTTTATTT
Sdhb	GACGTCAGGAGCCAAAATGG	CTCGACAGGCCTGAAACTGC
Tgfb1	*GCTGTACATTGACTTTAGGAAGGA*	*AGGGTCCCAGACAGAAGTT*
Tgfbr1	*GGGCTTAGTGTTCTGGGAAA*	*CCGATGGATCAGAAGGTACAAG*
Tlr2	*CCAAAGAGCTCGTAGCATCC*	*AGGGGCTTCACTTCTCTGCT*
Uqcrfs1	CAGTCGAAGTGTCCCAGTTAAG	CACAACCAAGATGAGTACAGACA
Uqcrq	TTCAGCAAAGGCATCCCCAA	CGACTGCTCAAACTCCTGGT
Pparg-ChIP	CTCAGTGTGGGAACCAAGATTA	GCTACAGAGGAAGAGGAATGAC
Atp5l-ChIP	CACCCATTCACCGACTTCTT	CAGCTCCTACTGGCTTGTTT
Cox5a-ChIP	GGCCCTGCATTGCTAGATAA	GACACATACCACAACCAAACAAA
Cox5b-ChIP	GTGGCTAACAGGCATAACAAAC	GGCTTCCACCACGAATCATA
Cox6a1-ChIP	CAGGCAGCCAAGATCCTAAA	CCTCCAGGCATCCGTTATTG
Ndufa4-ChIP	CGAGCTATACTGGACGAAAGTG	AAGGGATCGTCATCGTGTAATC
Sdhb-ChIP	GGAAGAGAGAACAGAAAGACAGAG	CGGAGTACAACTAGCAGGATTT
Uqcrfs1-ChIP	AGACAGAGAGGCAGGATTAGA	TACCTACTTGGCACTGCTTG
Uqcrq-ChIP	ACCAGGCCTTTGCATAACA	GAGTAAGGGACAGCAGAAGAAC

Supplementary Table 2: Primers used for qRT-PCR and ChIP-qPCR

Supplementary Table 3

**Primers**	**Sequence (5'→3')**
**multiplexing adapter-top**	GATCGGAAGAGCACACGTCT
**multiplexing adapter-bottom**	ACACTCTTTCCCTACACGACGCTCTTCCGATCT
**MultipPCR Primer1.0**	AATGATACGGCGACCACCGAGATCTACACTCTTTCCCTACACG ACGCTCTTCCGATC*T
**Primer2- IndexM1-64**	CAAGCAGAAGACGGCATACGAGATCGTGATGTGACTGGAGTTC AGACGTGTGCTCTTCCGATC*T
**Primer2- IndexM2-64**	CAAGCAGAAGACGGCATACGAGATACATCGGTGACTGGAGTTC AGACGTGTGCTCTTCCGATC*T
**Primer2- IndexM3-64**	CAAGCAGAAGACGGCATACGAGATGCCTAAGTGACTGGAGTT CAGACGTGTGCTCTTCCGATC*T
**Primer2- IndexM4-64**	CAAGCAGAAGACGGCATACGAGATTGGTCAGTGACTGGAGTT CAGACGTGTGCTCTTCCGATC*T
**Primer2- IndexM5-64**	CAAGCAGAAGACGGCATACGAGATCACTGTGTGACTGGAGTT CAGACGTGTGCTCTTCCGATC*T
**Primer2- IndexM6-64**	CAAGCAGAAGACGGCATACGAGATATTGGCGTGACTGGAGTTC AGACGTGTGCTCTTCCGATC*T
**Primer2- IndexM7-64**	CAAGCAGAAGACGGCATACGAGATGATCTGGTGACTGGAGTTC AGACGTGTGCTCTTCCGATC*T
**Primer2- IndexM8-64**	CAAGCAGAAGACGGCATACGAGATTCAAGTGTGACTGGAGTT CAGACGTGTGCTCTTCCGATC*T
**Primer2- IndexM9-64**	CAAGCAGAAGACGGCATACGAGATCTGATCGTGACTGGAGTTC AGACGTGTGCTCTTCCGATC*T
**Primer2- IndexM10-64**	CAAGCAGAAGACGGCATACGAGATAAGCTAGTGACTGGAGTT CAGACGTGTGCTCTTCCGATC*T
**Primer2- IndexM11-64**	CAAGCAGAAGACGGCATACGAGATGTAGCCGTGACTGGAGTT CAGACGTGTGCTCTTCCGATC*T
**Primer2- IndexM12-64**	CAAGCAGAAGACGGCATACGAGATTACAAGGTGACTGGAGTT CAGACGTGTGCTCTTCCGATC*T

Supplementary Table 3: Primers used for RNA sequencing
