# Supplementary material for: Histone deacetylase 3 controls lung alveolar macrophage development and homeostasis
Source: Nat Commun. 2020 Jul 30;11:3822. doi: 10.1038/s41467-020-17630-6 (PMC7393351; doi:10.1038/s41467-020-17630-6)
Supplement: Supplementary file 2 — Description of Additional Supplementary Information [file 41467_2020_17630_MOESM2_ESM.pdf]

Description of Additional Supplementary Files

File Name: Supplementary Data 1
Description: Differentially expressed genes in preAMs at E18.5 from bulk RNA-seq.

File Name: Supplementary Data 2
Description: Gene ontology analysis of differentially expressed genes in preAMs from bulk RNA-seq.

File Name: Supplementary Data 3
Description: HDAC3-binding genes in AMs from ChIP-seq.
