# Supplementary material for: Histone deacetylase 3 controls lung alveolar macrophage development and homeostasis
Source: Nat Commun. 2020 Jul 30;11:3822. doi: 10.1038/s41467-020-17630-6 (PMC7393351; doi:10.1038/s41467-020-17630-6)
Supplement: Supplementary file 6 — Reporting Summary [file 41467_2020_17630_MOESM6_ESM.pdf]

nature research

Corresponding author(s): Mi, Q-S

Last updated by author(s): Jun 30, 2020

Reporting Summary

Nature Research wishes to improve the reproducibility of the work that we publish. This form provides structure for consistency and transparency in reporting. For further information on Nature Research policies, see our Editorial Policies and the Editorial Policy Checklist.

Statistics

For all statistical analyses, confirm that the following items are present in the figure legend, table legend, main text, or Methods section.

n/a	Confirmed	
☐	☒	The exact sample size (*n*) for each experimental group/condition, given as a discrete number and unit of measurement
☐	☒	A statement on whether measurements were taken from distinct samples or whether the same sample was measured repeatedly
☐	☒	The statistical test(s) used AND whether they are one- or two-sided<br>*Only common tests should be described solely by name; describe more complex techniques in the Methods section.*
☒	☐	A description of all covariates tested
☐	☒	A description of any assumptions or corrections, such as tests of normality and adjustment for multiple comparisons
☐	☒	A full description of the statistical parameters including central tendency (e.g. means) or other basic estimates (e.g. regression coefficient) AND variation (e.g. standard deviation) or associated estimates of uncertainty (e.g. confidence intervals)
☐	☒	For null hypothesis testing, the test statistic (e.g. *F*, *t*, *r*) with confidence intervals, effect sizes, degrees of freedom and *P* value noted<br>*Give P values as exact values whenever suitable.*
☒	☐	For Bayesian analysis, information on the choice of priors and Markov chain Monte Carlo settings
☒	☐	For hierarchical and complex designs, identification of the appropriate level for tests and full reporting of outcomes
☒	☐	Estimates of effect sizes (e.g. Cohen's *d*, Pearson's *r*), indicating how they were calculated

Our web collection on statistics for biologists contains articles on many of the points above.

Software and code

Policy information about availability of computer code

Data collection	BD FACSDiva software version 8.0.2 for FACS; QuantStudio 7 Flex Real-Time PCR System software version 1.2 for qRT-PCR; FSX-BSW (03.02.12) software for immunofluorescence.
Data analysis	FlowJo 10.5.3 for FACS results; GraphPad Prism 8.4.3 for statistics; Microsoft Excel 2016 for qRT-PCR results, cellSens Dimensions Imaging Software version 1.15 for image processing; Biomedical Genomics Workbench 5.0 for bulk RNA-seq reads alignment; DAVID Bioinformatics Resources 6.8 for GO analysis; GSEA 4.0.3 for gene set enrichment analysis; Ingenuity Pathway Analysis version 42012434 for pathway analysis; FastQC version v0.10.1 for sequencing read quality control, BBDuk (v36.19) for adapter trimming, seqtk (v1.2-r94, https://github.com/lh3/seqtk) for low-quality base trimming, Bowtie2 (v2.2.5) for read alignment, MACS2 for peak calling, ChIPseeker (v1.24.0) for peak annotation; 10× Cell RangerTM [v2.1] pipeline for scRNA-seq reads alignment, R-3.3.2 package Seurat's Canonical Correlation Analysis (CCA) workflow for scRNA-seq dataset analysis.

For manuscripts utilizing custom algorithms or software that are central to the research but not yet described in published literature, software must be made available to editors and reviewers. We strongly encourage code deposition in a community repository (e.g. GitHub). See the Nature Research guidelines for submitting code & software for further information.

Data

Policy information about availability of data

All manuscripts must include a data availability statement. This statement should provide the following information, where applicable:

- Accession codes, unique identifiers, or web links for publicly available datasets
- A list of figures that have associated raw data
- A description of any restrictions on data availability

All ChIP-seq, bulk RNA-seq, and scRNA-seq data reported here have been deposited in the Gene Expression Omnibus (GEO) under accession number SuperSeries

GSE122533. The data of PPAR-γ microarray (GSE602497, https://www.ncbi.nlm.nih.gov/geo/query/acc.cgi?acc=GSE60249) had been previously disclosed in GEO by other researches. DAVID Bioinformatics Resources 6.860 (https://david.ncifcrf.gov/) and RPG: Ribosomal Protein Gene database70 (http://ribosome.med.miyazaki-u.ac.jp/) are both publicly accessible. The source data underlying Figs. 1a-c, e, f, h, j, 2a-f, 3b, 4b, e, f, 5a, f, g, j, k, 6e, h, 7a, b, f, g, j, Supplementary Figs. 2a, b, 3a, b, 4a, b, 6a-c, 9 are provided in the Source Data file. The data supporting this study are available in the Article, Supplementary Information, Source Data or available from the authors upon reasonable requests. Source data are provided with this paper. The reporting summary and editorial checklist for this article are available as a Supplementary file.

Field-specific reporting

Please select the one below that is the best fit for your research. If you are not sure, read the appropriate sections before making your selection.

- [x] Life sciences
- [ ] Behavioural & social sciences
- [ ] Ecological, evolutionary & environmental sciences

For a reference copy of the document with all sections, see nature.com/documents/nr-reporting-summary-flat.pdf

Life sciences study design

All studies must disclose on these points even when the disclosure is negative.

Sample size	No statistical method was used to predetermine sample size. Sample size was chosen based on our prior studies using the same types of assays, as well as published literature, to ensure statistically significant results. Both in vitro and in vivo studies were performed with at least three biologically independent samples per group. Statistical tests then performed using GraphPad to provide confidence in the conclusions made.
Data exclusions	No data were excluded.
Replication	Bulk RNA-seq (three biologically independent samples/group), scRNA-seq (single sample/group), and ChIP-seq (single sample/group) were performed one time. Immunofluorescence, bone marrow chimera, ChIP-qPCR and qRT-PCR experiments were performed twice independently with similar results. All other experiments were performed at least three times independently with similar results.
Randomization	Mice and samples were randomly allocated to each analysis group at the start of experiment.
Blinding	Blinding was performed by removal of identifying information from each sample while primary data was collected during an experiment. Once data had been collected, blinding was not required for statistical or bioinformatic analysis as objective readouts had been used in all experiments and all samples were analyzed using an identical method for each experiment.

Reporting for specific materials, systems and methods

We require information from authors about some types of materials, experimental systems and methods used in many studies. Here, indicate whether each material, system or method listed is relevant to your study. If you are not sure if a list item applies to your research, read the appropriate section before selecting a response.

Materials & experimental systems

n/a	Involved in the study	
☐	☒	Antibodies
☐	☒	Eukaryotic cell lines
☒	☐	Palaeontology and archaeology
☐	☒	Animals and other organisms
☒	☐	Human research participants
☒	☐	Clinical data
☒	☐	Dual use research of concern

Methods

n/a	Involved in the study	
☐	☒	ChIP-seq
☐	☒	Flow cytometry
☒	☐	MRI-based neuroimaging

Antibodies

Antibodies used	The list of antibodies used for flow cytometry has been provided in the Supplementary Table 4.<br><br>The antibody used for ChIP-seq: anti-HDAC3 (Abcam, #ab7030, polyclonal, 2 μg/500 μl assay buffer).<br><br>The antibodies used for ChIP-qPCR: anti-RNA Polymerase II antibody (Millipore, #05-623B, clone CTD4H8, 1 μg/ml), anti-HDAC3 antibody (Abcam, #ab32369, clone Y415, 2 μg/ml), anti-PPAR-γ antibody (Cell Signaling, clone 81B8, 1 μg/ml), and rabbit IgG (Abcam, #ab171870, polyclonal, 1 μg/ml)
Validation	All of the listed antibodies used for flow cytometry have been validated using mouse primary cells. F4/80-PE and SiglecF-PE antibodies have also been validated by Immunofluorescence using mouse primary tissues. Anti-HDAC3 antibody for ChIP-seq was validated by the service vendor using Pre-Sure ChIP Antibody Validation Kit (EpiGentek, Cat. #P-2031). All antibodies used for ChIP-qPCR were validated by manufacturers for the applications and species used in this study and were supported by publications or

internal validation. See manufacturers websites for validation statements (https://www.emdmillipore.com/; https://www.abcam.com/; https://www.cellsignal.com/) found in technical data sheets.

Eukaryotic cell lines

Policy information about cell lines

Cell line source(s)	mouse alveolar macrophage MH-S cell line provided by ATCC.
Authentication	Growth properties, morphology, species determination (COI assay), and sterility test by ATCC.
Mycoplasma contamination	MH-S cell line has been tested negative for mycoplasma contamination by ATCC.
Commonly misidentified lines (See ICLAC register)	No misidentified cell line was used.

Animals and other organisms

Policy information about studies involving animals; ARRIVE guidelines recommended for reporting animal research

Laboratory animals	Hdac3fl/fl mice were provided by Scott W. Hiebert54. C57BL/6 (Strain #000664), B6.SJL (Strain #002014), Cd11cCre (Strain #008068), Csf1ricre (Strain #021024), UbcCreER (strain #007001), and Ppargfl/fl (strain #004584) mice were purchased from the Jackson Laboratory (Bar Harbour, ME). To generate myeloid-lineage-specific HDAC3 mutant mice, we crossed Hdac3fl/fl and Csf1ricre mice (back to a B6/C57 mouse genetic background for 6 generations). To generate CD11c-expressing, cell-specific HDAC3 mutant mice, we crossed Hdac3fl/fl and Cd11cCre mice. To generate inducible HDAC3 deletion mice, we crossed Hdac3fl/fl and UbcCreER mice. To generate myeloid lineage-specific PPAR-γ-deficient mice, we crossed Ppargfl/fl and Csf1ricre mice. To generate heterozygous mice expressing both CD45.1 and CD45.2, named B6.SJLhetero, we crossed B6.SJL (CD45.1) with C57BL/6 (CD45.2) mice.<br><br>Male and female mice from 6 – 18 weeks of age were used. All experiments included age- and sex-matched littermate controls. Embryonic development was estimated considering the day of vaginal plug formation as embryonic age of 0.5 days.
Wild animals	No wild animals were used.
Field-collected samples	No field-collected samples were used.
Ethics oversight	All mice were housed under specific pathogen-free conditions at temperatures of 20-26°C with 30-70% humidity and a 12-hour light/12-hour dark cycle at Henry Ford Health System. Experimental animal protocols were performed in accordance with the guidelines of the Institutional Animal Care and Use Committee.

Note that full information on the approval of the study protocol must also be provided in the manuscript.

ChIP-seq

Data deposition

☒ Confirm that both raw and final processed data have been deposited in a public database such as GEO.

☒ Confirm that you have deposited or provided access to graph files (e.g. BED files) for the called peaks.

Data access links<br>*May remain private before publication.*	The ChIP-seq, bulk RNA-seq, and scRNA-seq data reported here have been deposited in the Gene Expression Omnibus (GEO) under accession number SuperSeries GSE122533 (https://www.ncbi.nlm.nih.gov/geo/query/acc.cgi?acc=GSE122533).
Files in database submission	GSE122393, GSE122529, GSE122532
Genome browser session (e.g. UCSC)	No longer applicable.

Methodology

Replicates	No replicates for each ChIP.
Sequencing depth	WT HDAC3ChIPseq: 24 million total reads, 3,631,196 uniquely mapped reads, 50 bp lengths of reads, single-end;<br>KO HDAC3 ChIPseq: 19 million total reads, 572,642 uniquely mapped reads, 50 bp lengths of reads, single-end.<br>WT H3K27ac ChIPseq: 27 million total reads, 1,414,313 uniquely mapped reads, 50 bp lengths of reads, single-end.<br>KO H3K27ac ChIPseq: 25 million total reads, 6,496,699 uniquely mapped reads, 50 bp lengths of reads, single-end.<br>WT H3K9ac ChIPseq: 18 million total reads, 979,027 uniquely mapped reads, 50 bp lengths of reads, single-end.<br>KO H3K9ac ChIPseq: 16 million total reads, 1,863,641 uniquely mapped reads, 50 bp lengths of reads, single-end.
Antibodies	anti-HDAC3 (Abcam, #ab7030, polyclonal, 2 µg/500 µl assay buffer).
Peak calling parameters	macs2 callpeak -f BAM -g mm -B -q 0.01<br>macs2 bdgcmp -m FE

Data quality

Raw ChIP-seq reads were quality checked using the software FastQC version v0.10.1 and processed using BBDuk (v36.19) and seqtk (v1.2-r94, https://github.com/lh3/seqtk) to trim the adapters and low-quality bases, respectively. The trimmed reads were then aligned to the mouse mm10 genome sequence using Bowtie2 (v2.2.5) and only uniquely matching reads were retained. Mapping results of each ChIP and the input sample were subjected to ChIP enriched peak calling using the MACS2. A total of 29,201 and 2,775 peaks for WT- and KO-HDAC3, 7,509 and 2,659 peaks for WT- and KO-H3K27ac, 3,418 and 6,448 peaks for WT- and KO-H3K9ac, respectively, are at FDR 5% and above 5-fold enrichment.

Software

FastQC version v0.10.1 for sequencing read quality control, BBDuk (v36.19) for adapter trimming, seqtk (v1.2-r94, https://github.com/lh3/seqtk) for low-quality base trimming, Bowtie2 (v2.2.5) for read alignment, MACS2 for peak calling, ChIPseeker (v1.24.0) for peak annotation.

Flow Cytometry

Plots

Confirm that:

- [x] The axis labels state the marker and fluorochrome used (e.g. CD4-FITC).
- [x] The axis scales are clearly visible. Include numbers along axes only for bottom left plot of group (a 'group' is an analysis of identical markers).
- [x] All plots are contour plots with outliers or pseudocolor plots.
- [x] A numerical value for number of cells or percentage (with statistics) is provided.

Methodology

Sample preparation

Pregnant females were sacrificed by CO2 exposure. Embryos ranging from embryonic days E10.5-E18.5 were removed from the uterus and washed in 4°C phosphate-buffered saline ([PBS], Invitrogen, Carlsbad, CA). Embryos were exsanguinated through decapitation. Lungs, liver, brain, kidneys, spleen, and pancreas were collected, minced into tiny pieces, and incubated for 30 min in PBS containing 1 mg/ml collagenase D (Roche, Basel, Switzerland), 0.01% DNase I (Worthington Biochemical Corp., Lakewood, NJ), and 3% FBS (Hyclone, San Angelo, TX) at 37°C. Erythrocytes from all the samples were lysed for 3 min with 0.83% NH4Cl buffer. The cells from the adult tissues were isolated by the same method with some modifications. The adult lungs were harvested, minced, and incubated in PBS containing 1 mg/ml collagenase D (Roche), 0.01% DNase I (Worthington), and 3% FBS (Hyclone) at 37°C for 45 min. The erythrocytes were lysed as described above. All the cell suspensions were passed through a 70 μm cell strainer (BD Biosciences, San Jose, CA).

Instrument

FACSAria™ II or FACSCelesta™ flow cytometer (BD Biosciences)

Software

BD FACSDiva software version 8.0.2 for data collection and FlowJo 10.5.3 (BD Biosciences) for data analysis.

Cell population abundance

The purity of the isolated populations was >95%, which was analyzed by acquiring the post-sort samples on the flow cytometer.

Gating strategy

Single-cell suspensions of lung and other indicated organs were prepared from Hdac3fl/fl embryos at E18.5. After excluding dead cells (FSC-A vs. SSC-A) and doublets (FSC-A vs. FSC-H), all cells were gated based on CD45 expression. CD45+ cells were further gated based on F4/80 and CD11b expression for preAMs (F4/80intCD11bint), TRMs (F4/80hiCD11bint), and monocytes (F4/80intCD11bhi). CD117 and F4/80 expression was analyzed for EMPs (CD117hiF4/80–), pMacs (CD117–F4/80–), and macrophages (CD117–F4/80+) within CD45+Lin– live singlets in YS at E9.5. EMPs were also analyzed within CD45+ live cells in FL at E12.5. FL monocytes (CD64loCD11bhiLy6chi) were analyzed within CD45+ live cells at E18.5. >97% of CD64loCD11bhi cells were Ly6chi. A similar gating strategy was used to sort and analyze lung AMs (CD11chiSiglec Fhi) within CD45+ live cells from Hdac3fl/fl adults. Representative data shown are from at least 3 independent experiments.

- [x] Tick this box to confirm that a figure exemplifying the gating strategy is provided in the Supplementary Information.
